# Output from VIP cells of the mammalian central clock regulates daily physiological rhythms

**DOI:** 10.1038/s41467-020-15277-x

**Published:** 2020-03-19

**Authors:** Sarika Paul, Lydia Hanna, Court Harding, Edward A. Hayter, Lauren Walmsley, David A. Bechtold, Timothy M. Brown

**Affiliations:** 1 Centre for Biological timing, Faculty of Biology Medicine & Health, University of Manchester, Manchester, UK; 2 School of Pharmacy, University of Reading, Reading, UK

**Keywords:** Circadian regulation, Neural circuits

## Abstract

The suprachiasmatic nucleus (SCN) circadian clock is critical for optimising daily cycles in mammalian physiology and behaviour. The roles of the various SCN cell types in communicating timing information to downstream physiological systems remain incompletely understood, however. In particular, while vasoactive intestinal polypeptide (VIP) signalling is essential for SCN function and whole animal circadian rhythmicity, the specific contributions of VIP cell output to physiological control remains uncertain. Here we reveal a key role for SCN VIP cells in central clock output. Using multielectrode recording and optogenetic manipulations, we show that VIP neurons provide coordinated daily waves of GABAergic input to target cells across the paraventricular hypothalamus and ventral thalamus, supressing their activity during the mid to late day. Using chemogenetic manipulation, we further demonstrate specific roles for this circuitry in the daily control of heart rate and corticosterone secretion, collectively establishing SCN VIP cells as influential regulators of physiological timing.

## Abstract (web summary)

VIP-expressing neurons play a central role in circadian timekeeping within the mammalian central clock. Here the authors use opto- and chemogenetic approaches to show that VIP neuronal activity regulates rhythmic activity in downstream hypothalamic target neurons and their physiological functions.

## Introduction

The ability to adjust physiology and behaviour in anticipation of daily changes in the environment is critical for survival. Mammals achieve this via a master circadian clock in the suprachiasmatic nucleus (SCN) whose role is to coordinate rhythms in cells and tissue function throughout the body according to predictable individual daily variations in demand^1–3^.

The SCN comprises a functionally and neuroanatomically heterogeneous network of neurons, many of which possess intrinsic timekeeping capabilities, receive input from the retina and/or other SCN neurons, collectively resulting in robust daily variations in electrophysiological output^4,5^. However, despite remarkable progress in unravelling the basic biological machinery underpinning SCN timekeeping, the mechanisms by which rhythmic output from the central clock is used to differentially time the diverse physiological systems under circadian control remain a key question.

Subsets of SCN neurons directly innervate various nuclei important for neuroendocrine, homeostatic and autonomic control; principally the subparaventricular zone (SPZ), the paraventricular nuclei of the hypothalamus (PVN), dorsomedial hypothalamus, medial pre-optic area and paraventricular thalamus^6,7^. SCN cells can also be readily subdivided based on neuropeptide co-expression, with the best-studied sub-types (expressing arginine vasopressin—AVP or vasoactive intestinal polypeptide—VIP) known to provide differential innervation of these downstream target structures^7–10^. Accordingly, one promising hypothesis is that distinct SCN neuronal subpopulations may provide specifically timed daily output signals to control rhythms in different aspects of physiology and behaviour^11^.

While available data support the notion that subsets of SCN neurons could provide distinctly timed output signals^11–14^, attempts to link the function of identified neuron subtypes to specific aspects of physiological control have primarily focussed on AVP cells^11,15–17^. By contrast, while VIP is known to play vital roles in internal SCN timekeeping^18–21^, the specific contribution of VIP cell output to downstream physiological timing remains largely unexplored. Hence, while various studies have reported deficits in physiological/behavioural rhythms in mice lacking VIP or its receptor VPAC_2_^22–29^, the loss of this signalling pathway produces a global disruption of SCN rhythmicity^18–21^. These generalised impairments, therefore, obscure any specific roles for VIP cells in physiological control.

Here we employ large-scale multielectrode recording and targeted optogenetic manipulations to define the characteristic daily activity profiles of individual VIP neurons in the intact adult mouse SCN and determine how these influence rhythmic activity in downstream target cells. We then employ chemogenetic manipulations in freely-behaving mice to reveal specific roles for the VIP cell output circuitry in shaping rhythms of key clock-controlled neuroendocrine and physiological outputs.

## Results

### Properties of SCN VIP neurons

**Fig. 1:**
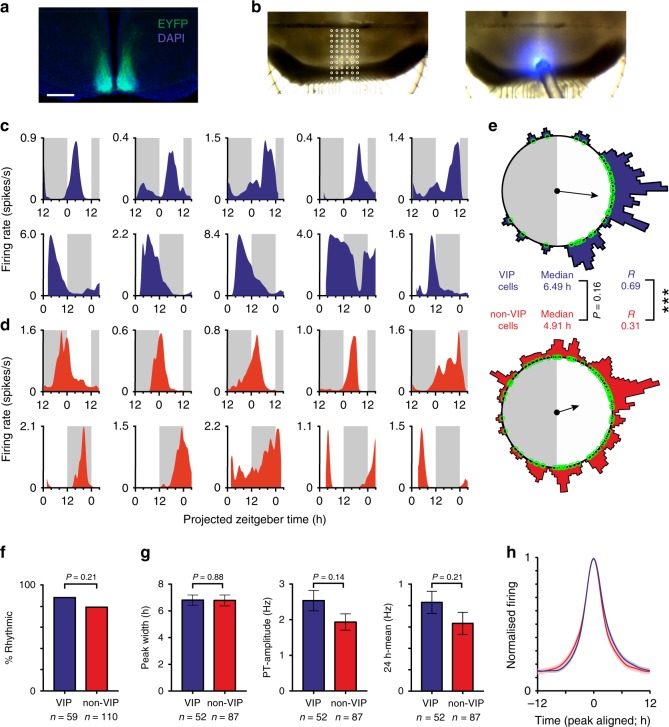
Suprachiasmatic nucleus VIP neurons provide a coordinated circadian output. **a** Antibody enhanced EYFP-signal from the SCN of a VIP-ChR2 mouse. Scale bar: 500 µm. **b** Light microscope images of a VIP-ChR2 SCN slice in situ on the pMEA; top: overlaid recording site locations, bottom: optical fibre positioning (460 nm stimulus; 792 mW/m^2^ at fibre tip). **c**, **d** Example spontaneous firing activity plots for optogenetically identified VIP (**c**) and non-VIP (**d**) SCN neurons from slices prepared during late (upper panels) or early (lower panels) projected day. **e** Rayleigh plots showing phase distribution for isolated VIP (blue) and non-VIP (red) SCN neurons that exhibited circadian variation in firing (*n* = 52 and 87 cells, respectively, from 16 slices). Individual cells are represented by open circles, external histograms represent relative population density (Gaussian smoothing; σ = 10 min). Phase and clustering strength compared by multilevel mixed-effects model (with slice preparation included as a 2^nd^ level random effect) and Browne–Forsythe’s tests, respectively on linearised data (see Methods). **f** Proportion of isolated VIP and non-VIP SCN neurons displaying evidence of circadian variation in firing, compared by Fisher's exact test. **g** Peak width (full width at half-max firing; left), Peak-trough amplitude for firing rhythm (middle) and 24 h mean firing rate (right) for rhythmic VIP and non-VIP SCN neurons. Data compared by multilevel mixed-effects linear model as above. **h** Normalised, peak aligned, 24-h firing profiles of rhythmic VIP and non-VIP neurons, illustrating the very similar circadian waveforms. *P < 0.05, ***P < 0.001.

While SCN VIP neurons are now known to exhibit robust circadian timekeeping properties^30–33^, the daily electrophysiological profiles of individual SCN neurons in the intact adult SCN and their relationship to whole-animal physiological rhythms remain unknown. To address this, we employed a mouse line^30,34^ where VIP cells express the light-gated cation channel, channelrhodopsin2 (*Vip*^*+/cre*^; *Ai32*^*+/*−^, termed here VIP-ChR2; Fig. 1a) and performed perforated multielectrode array (pMEA) recordings from the SCN of acutely prepared adult mouse brain slices (Fig. 1b).

Initially, we evaluated the impact of brief (0.3–3 ms) blue light flashes via an optical fibre positioned over the SCN during pMEA recordings across a full circadian cycle ex vivo. Under these conditions, the number of pMEA recording sites where we observed excitatory responses increased as a function of flash duration, with sites located further from the flash centre (and therefore receiving less light) requiring longer durations to reach firing threshold (Supplementary Fig. 1a–c). This effect plateaued for ≥2 ms flashes, where the distribution of electrode sites showing optogenetic responses matched that expected for SCN VIP cells^30–32,34,35^. Accordingly, the observed response latencies (mean ± SEM: 1.9 ± 0.1 ms; *n* = 98 responding sites) were similar those reported previously for direct activation in patch recordings^34^. As a result of this near synchronous activation of VIP neurons, single unit isolation from these population recordings was challenging. As such, subsequent experiments employed a ‘ramped’ stimulus (Supplementary Fig. 1d) comprising a train of multiple brief flashes to de-correlate the activation of individual VIP neurons. As predicted, these allowed us to readily isolate optogenetically activated neurons across appropriately located SCN recording sites (Supplementary Figs. 1d, e and 2a–c). Owing to the use of these ramped pulses, response latencies were, however, longer than for simple flashes (mean ± SEM: 6.9 ± 0.7 ms; *n* = 31 activated neurons from 4 slices).

To confirm that the observed responses reflected a direct ChR2-mediated excitation, we then applied antagonists of the main signalling pathways utilised by VIP neurons. Excitatory optogenetic responses persisted unchanged during application of a VIP receptor antagonist^36^ and the GABA_A_ receptor locker (+)-bicuculline (Supplementary Fig. 1e, g), ruling out possible synaptic mechanisms. By contrast, among rarer cells displaying optogenetically-driven inhibition, responses persisted following application of VIP antagonist but were subsequently abolished under GABA_A_-receptor blockade (Supplementary Fig. 1f, h), confirming a synaptically-mediated origin. Given the lack of VIP antagonist effect in these experiments, we also validated the sufficiency of this manipulation for preventing exogenous VIP-driven responses. As expected^37^, bath application of 100 nM VIP evoked excitatory or inhibitory responses in subsets of SCN neurons which, in both cases, were reliably prevented by antagonist pre-treatment (Supplementary Fig. 2d–g). As reported previously then^34^, the optical stimuli employed here do not drive any noticeable VIP-receptor mediated responses in VIP-ChR2 mice.

Having established an approach for reliable identification of VIP neurons, we employed ramped stimuli during long-term pMEA recordings from VIP-ChR2 SCN slices (1pulse/min over ≥26 h). From 16 recordings (slices prepared early or late day, *n* = 8/time-point), 59 neurons were clearly identified as VIP cells based on optogenetic response (Supplementary Fig. 3a). The majority of these VIP cells (*n* = 52/59) displayed robust circadian variation in spontaneous activity (see methods), with peak firing strongly clustered across mid-late projected day (Fig. 1c, e, f). These peak firing times were independent of the time of slice preparation (median ZT 6.0 vs. 6.6 h for early vs. late day slices, *P* = 0.5 from modified-linear multilevel mixed-effects model; *n* = 35 and 17 respectively), consistent with previous data indicating that acute electrophysiological recordings of this type reliably reflect SCN rhythms in the intact animal^14,19,38–40^.

We also identified many SCN cells that lacked rapid excitatory responses to optogenetic stimulation (Supplementary Fig. 3b), the majority which (*n* = 87/110 non-VIP cells) also exhibited circadian variations in spontaneous firing (Fig. 1d–f). Indeed, the basic properties of VIP and non-VIP neurons were very similar (Fig. 1f–h). Importantly, however, there were differences in the timing of activity across the two populations. In particular, while peak VIP cell activity was strongly clustered across the mid-late projected day, the non-VIP population exhibited a significantly broader distribution of peak times that was skewed towards earlier portions of the day (Fig. 1e). Analysis of within-slice estimates of clustering and phasing for the two subpopulations produced qualitatively similar results (Supplementary Fig. 3c–f). Thus, our data indicate that VIP-expressing neurons constitute a functionally distinct subgroup within the SCN, whose output primarily occupies a specific temporal window across the mid-late day.

To confirm that our experimental procedures did not alter the timing of SCN cell activity, we also compared overall SCN population output to equivalent data from control mice (*VIP*^*+/+*^*; Ai32*^*+/*−^; Supplementary Fig. 3a). Importantly, there were no significant differences in the phase or clustering of SCN cellular rhythms, the proportion of neurons showing circadian variation nor the single cell circadian waveforms (Supplementary Fig. 4a–d). We did, however, find that absolute amplitudes of VIP-ChR2 SCN firing rate rhythms were modestly reduced relative to control (Supplementary Fig. 4e), perhaps reflecting the reduced VIP expression observed in *VIP*^*+/cre*^ mice^41^. Notably, circadian rhythms in behaviour are not noticeably impaired in this line, a finding which is consistent with our own observations that the basic rhythmic properties are intact in VIP-ChR2 slices.

### Properties of VIP target neurons

**Fig. 2:**
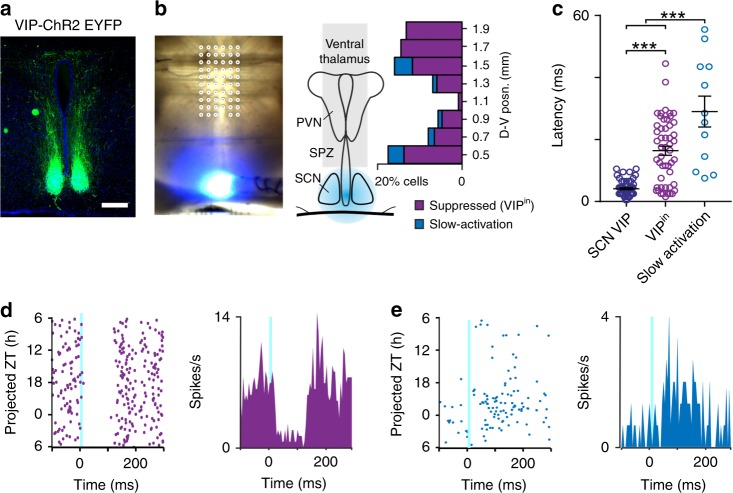
Optogenetic identification of VIP^in^ cells in regions receiving SCN input. **a** Immunohistochemical amplification of ChR2-EYFP expression from a VIP-ChR2 mouse, showing terminal fields of the VIP neurons lying within the SPZ, PVN and ventral thalamus. **b** Light microscope images of a VIP-ChR2 brain slice on the pMEA with overlaid recording site locations (left) and schematic showing the percentage of identified cells displaying suppressions or slow-activation following 10 ms light flashes (right; *n* = 51 and 12 cells, respectively from 718 isolated neurons). **c** Response latencies for SCN VIP cells and SPZ, PVN and ventral thalamic cells exhibiting optogenetic-driven suppressions (VIP^in^) or slow-activation. Data analysed by multilevel mixed-effects linear model (F_2,44.89_ = 18.0, *P* < 0.001) with Tukey post-tests. **d**, **e** Flash evoked single unit spike rasters over 24 h (left) and mean response histograms (right) for representative cells showing optogenetic-driven suppression (**d**) or slow-activation (**e**).

We next sought to identify downstream neurons that received input from SCN VIP cells. Accordingly, we evaluated data from long-term (≥26 h) pMEA recordings spanning known target regions (SPZ, PVN, and ventral thalamus) while optogenetically stimulating the SCN (Fig. 2a, b; *n* = 44 VIP-ChR2 slices). Across these targets, we identified a subset of neurons (herein termed VIP^in^) that exhibited rapid attenuation of firing following optogenetic stimulation, consistent with inhibitory input from SCN VIP neurons (Fig. 2b–d; *n* = 51 cells). More rarely, neurons exhibiting modest increases in firing in response to optogenetic stimulation of the SCN were also identified (Fig. 2b, e; *n* = 12/718 total cells). Such responses were qualitatively different from those of SCN VIP cells, exhibiting sluggish kinetics indicative of a polysynaptic origin (Fig. 2c; referred to here as slow-activation). Collectively then, these data indicate that VIP cells convey primarily inhibitory signals to downstream neurons.

**Fig. 3:**
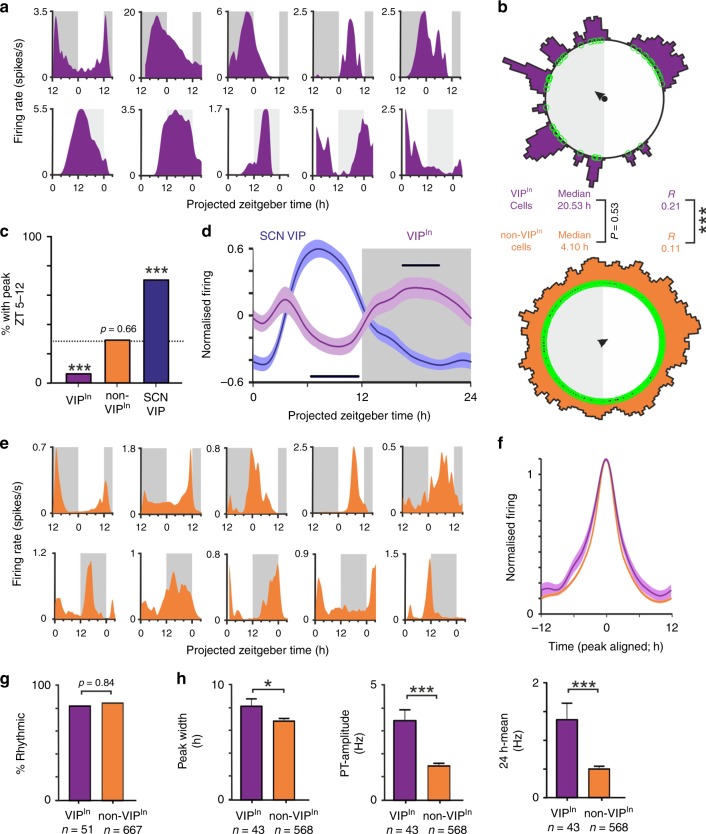
VIP^in^ cells exhibit robust daily variations in electrophysiological output. **a**, **e** Example spontaneous firing activity from optogenetically identified VIP^in^ (**a**) and non-VIP^in^ cells (**e**), from slices prepared during late (top panels) or early (bottom panels) projected day. **b** Rayleigh plots showing phase distribution for isolated VIP^in^ and non-VIP^in^ cells that exhibited evidence of circadian variation in firing (*n* = 43 and 567 cells, from 22 late and 23 early day slices, respectively). Conventions and analysis as in Fig. 1e. **c** Proportion of VIP^in^, non-VIP^in^ and SCN VIP cells with peak firing between projected ZT5-12, dotted line represents proportion expected by chance, data analysed by Fisher's exact test. **d** Mean ± SEM normalised, ZT aligned, 24 h firing profiles of rhythmic VIP^in^ and SCN VIP neurons. Black bars indicate when VIP^in^ activity is significantly different from the daily mean. Data analysed by one-way RM ANOVA (F_3.169, 133.1_ = 3.42, *P* = 0.02) with post-hoc one-sample *t*-tests (*P* > 0.05). **f** Mean ± SEM normalised, peak aligned, 24 h firing profiles of rhythmic VIP^in^ and non-VIP^in^ neurons. **g** Proportion of isolated VIP^in^ and non-VIP^in^ cells d^i^splaying evidence of circadian variation in firing, proportions compared by Fisher's exact test. **h** Peak width, Peak-trough amplitude (middle) and 24 h mean firing rate (right) for rhythmic VIP^in^ and non-VIP^in^ cells. Data compared by multilevel mixed-effects linear model. *P < 0.05, ***P < 0.001.

Since SCN VIP cells exhibit maximal firing across the mid-late subjective day, we next asked whether this translated to an antiphase rhythm in firing for the VIP^in^ cells that receive this signal. Accordingly, the majority of VIP^in^ cells (*n* = 43/51) displayed clear evidence of circadian variation in spontaneous firing, with peak firing times distributed across the projected night and early projected day (Fig. 3b–d; Supplementary Fig. 5a). The phase clustering in VIP^in^ cells was weaker than that of the SCN VIP cells themselves (Rayleigh *R* = 0.21 vs. 0.69, *P* < 0.0001; Browne–Forsythe’s test) and was not overtly dependent on the anatomical location of the recorded neurons (including significant clusters in the SPZ region and around the dorsal cap of the PVN) or the time of slice preparation (Supplementary Fig. 5b–f). Nevertheless, collectively there was a clear absence of VIP^in^ cells whose peak firing occurred during the mid-late projected day (Fig. 3c) and, across the population, average firing rates fell significantly below the daily mean during this epoch (Fig. 3d). This decrease in VIP^in^ cell activity, which aligns with the daily peak in SCN VIP cell population firing, is therefore consistent with a rhythmic inhibitory drive imposed by the SCN VIP cells.

Interestingly, the majority of non-VIP^in^ cells identified in the same experiments (*n* = 568/667) also displayed daily variations in firing, although in this case we found cells exhibiting peak firing at all phases across the circadian cycle (Fig. 3b, d, e). Thus, across this population, phase clustering was significantly weaker than that observed for VIP^in^ cells (Fig. 3b), with many non-VIP cells exhibiting peak firing during the mid to late day (Fig. 3d). As expected from the lack of phase clustering in the aggregated cell population, within-slice phase estimates for non-VIP^in^ cells were similarly distributed throughout the circadian cycle (Rayleigh *R* = 0.1; *n* = 44 slices). There were also differences in the circadian properties of the non-VIP^in^ cells; specifically, peak widths were narrower and peak to trough amplitudes and mean firing rates lower than observed for VIP^in^ cells (Fig. 3g, h).

In sum, input from SCN VIP cells is specifically associated with high amplitude circadian variation in the firing of downstream target cells, which characteristically exhibit low activity during mid-late portions of the day and high activity during the night or early day.

### Impact of VIP cell output on target neurons

**Fig. 4:**
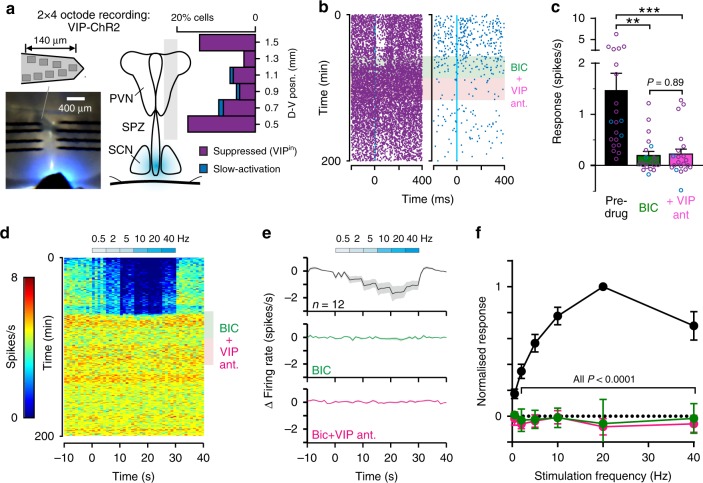
Suprachiasmatic nucleus VIP neurons drive GABA-mediated responses in downstream target neurons. **a** Slice image and schematics (left) illustrating 2 × 4 octode probe placement and position of fibre-optic delivered light flash. Right panel shows the percentage of identified cells displaying suppressions or slow-activation following 10 ms light flashes (in total *n* = 37 and 3 cells respectively from 561 isolated neurons), from ex vivo recordings across the SPZ, PVN and ventral thalamus of VIP-ChR2 mice. **b** Peri-event spike raster plots for representative VIP^in^ (supressed) and cells with slow-activation prior-to and following BIC application and subsequent co-application with VIP antagonist. **c** Mean ± SEM optogenetically-evoked change in firing rate of individual cells prior to and following antagonist treatment (*n* = 18 VIP^in^ and *n* = 3 cells with slow-activation). Data analysed by multilevel mixed-effects linear model (Treatment: F_2,24.521_ = 8.44, *P* = 0.002) with Tukey’s post-tests. **d**, **e** Pseudo-coloured spike raster for representative VIP^in^ cell (**d**) and mean ± SEM baseline-subtracted firing rates across the population (**e**; *n* = 12 cells) during exposure to repeated stimulation (10 ms flashes) at varying frequencies, prior to and following antagonist treatment as above. **f** Mean ± SEM normalised change in firing (relative to no stimulation) for VIP^in^ cells (*n* = 12) as a function of optogenetic stimulation frequency during pre-drug and antagonist treatment conditions. Data analysed by multilevel mixed-effects linear model (Frequency: F_5,21.785_ = 6.99, *P* < 0.001; Treatment: F_2,20.357_ = 49.72, *P* < 0.001; Interaction: F_10,23.401_ = 16.71, *P* < 0.001) with Sidak’s post-test. ***P < 0.001, **P < 0.01.

We next investigated the mechanisms by which activation of VIP neurons modulates the firing rate of VIP^in^ cells. For practical reasons, here we used penetrating multisite probes (rather than pMEA) to sample neurons across VIP cell target regions in ex vivo VIP-ChR2 slices while optogenetically stimulating the SCN (Fig. 4a). Data obtained using this closely approach matched the results of pMEA recordings, however. Hence, we again identified a subset of neurons across the VIP cell target regions that exhibited reliable decreases in firing following optogenetic stimulation (Fig. 4a; *n* = 37 VIP^in^) and a smaller subset that exhibited slow activations (*n* = 3/561 isolated neurons). Stimulus-driven decreases in VIP^in^ cell firing were prevented by (+)-bicuculline, with no further change in activity following co-application of the VIP antagonist (Fig. 4b, c; *n* = 19 VIP^in^ cells tested). Similarly, (+)-bicuculline attenuated responses in the 3 cells which exhibited slow-activation (Fig. 4b, c), consistent with the polysynaptic origin of these responses suggested above.

In sum, our data suggest that SCN VIP cells primarily influence the firing of their downstream target neurons via GABA rather than VIP signalling. Given recent data indicating that paired high-frequency stimuli are required for VIP-dependent phase shifts in VIP-ChR2 mice^30^, we next evaluated the impact of paired optogenetic stimulation at instantaneous frequencies spanning the range exhibited by VIP neurons (Supplementary Fig. 6a; 0.5–40 Hz). Across this range, VIP^in^ cell responses were reliably predicted by a linear sum of the response to low frequency (0.5 Hz) single pulse stimulation, as were responses of those showing slow-activation (Supplementary Fig. 6b, c). Moreover, in all cases tested, responses were essentially abolished by (+)-bicuculline treatment as for single pulse stimuli (Supplementary Fig. 6d, e).

We also examined the impact of sustained SCN VIP cell stimulation (Fig. 4d; 5s blocks of 0.5–40 Hz stimulation). As expected, this manipulation evoked a rate-dependent decrease in the steady-state firing of VIP^in^ cells (Fig. 4e). The maximal inhibition (57 ± 8% decrease in spontaneous firing) was consistently observed following stimulation at 20 Hz (Fig. 4e, f)—generally considered as the upper limit for spontaneous SCN cell firing^20^. As above, however, responses to sustained optogenetic stimulation were reliably abolished by (+)-bicuculline across all frequencies (Fig. 4f).

Our data, therefore, confirm that SCN VIP cells can exert a powerful influence over the activity of their downstream target neurons and that this effect is primarily driven by GABA rather than VIP signalling. Since, in our experiments (+)-bicuculline preceded VIP antagonist application, we do not exclude the possibility that VIP release could exert some modulatory influence over these GABA-dependent changes in VIP^in^ cell firing. Nonetheless, we also found that bath application of exogenous VIP exerted negligible effects on the firing rate of VIP^in^ and neighbouring non-VIP^in^ neurons (Supplementary Fig. 6f, g). Most likely then, the mouse SCN primarily employs VIP for internal communication rather than output (at least to those regions we recorded from), an idea supported by the paucity of VIP receptors across the recorded regions in mice^42,43^.

To establish how GABAergic output from VIP cells influences daily rhythms in their postsynaptic target neurons, we next assessed the impact of optogenetically inhibiting SCN VIP cell firing. Accordingly, we established a mouse line in which VIP cells express the light-gated proton pump Archaerhosopsin-3 (*VIP*^*+/cre*^; *Ai40*^*+/−*^; termed here VIP-Arch) and performed long-term pMEA recordings from the SCN and target regions (*n* = 23 slices).

**Fig. 5:**
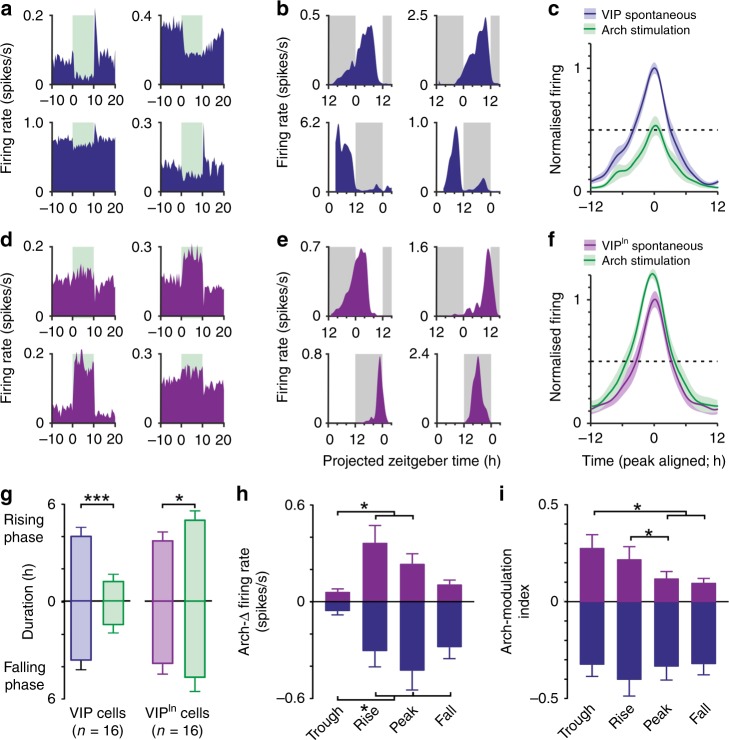
Optogenetic inhibition of SCN VIP cells modulates daily variations in VIP^in^ cell activity. **a**, **d** Peri-event histograms for example SCN VIP (**a**) and extra-SCN VIP^in^ (**d**) cells identified in pMEA recordings from VIP-Arch slices by 10s yellow light steps applied to the SCN region (565 nm, 140 mW/mm^2^ at fibre tip; means of ~300 trials). **b**, **e** Example spontaneous firing activity from optogenetically identified SCN VIP (**b**) and extra-SCN VIP^in^ cells (**e**), from slices prepared during late (top panels) or early (bottom panels) projected day (traces correspond to units in **a**, **d**). **c**, **f** Mean ± SEM normalised, peak aligned, 24 h firing profiles of SCN VIP (**c**) and extra-SCN VIP^in^ neurons (**f**) under baseline conditions and during Arch activation. **g** Mean ± SEM durations of rising and falling phase of the daily peak (time to <half-max) for SCN VIP and extra-SCN VIP^in^ neurons under baseline conditions and during Arch activation. Data for each class was analysed by multilevel mixed-effects linear model (VIP cells—Arch effect: F_1,43.72_ = 32.94, *P* > 0.001; Rising vs. falling phase: F_1,4.761_ = 0.03, *P* = 0.86; Interaction: F_1,43.72_ = 0.47, *P* = 0.50; VIP^in^ cells—Arch effect: F_1,36.58_ = 5.77, *P* = 0.02; Rising vs. falling phase: F_1,18.72_= 0.004, *P* = 0.95; Interaction: F_1,36.58_ = 0.22, *P* = 0.64). **h**, **i** Mean ± SEM Arch-driven change in firing rate (**h**) and firing rate modulation (**i**; (Arch-spontaneous)/(Arch+spontaneous)) for SCN VIP (blue) and extra-SCN VIP^in^ (purple) neurons across different quartiles of the cells’ daily firing rhythm. In both cases data for each class analysed by multilevel mixed-effects linear model (**h** VIP cells: F_3,38.66_ = 7.26, *P* = 0.001; VIP^in^ cells: F_3,21.88_ = 5.68, *P* = 0.005; **i** VIP cells: F_3,15.05_ = 0.30, *P* = 0.88; VIP^in^ cells: F_3,139.99_ = 4.67, *P* = 0.004).Tukey’s post-tests were applied where ANOVA reported a significant effect; *P < 0.05, ***P < 0.001.

As expected, illumination of VIP-Arch slices with yellow light (10 s steps) rapidly decreased firing of a subset of SCN neurons, typically followed by rebound excitation on termination (Fig. 5a; *n* = 16/67 SCN neurons). Just as in VIP-ChR2 mice, SCN VIP cells identified in this manner reliably exhibited peak spontaneous firing across the mid-late projected day (Fig. 5b). Similarly, recordings across VIP-cell target regions revealed a subset of neurons that exhibited rapid increases in firing in response to illumination of the SCN, as expected for a disinhibition of VIP^in^ cell activity (Fig. 5d; *n* = 16/248 cells). These VIP^in^ cells exhibited the expected circadian variations characterised by peak firing during the night or early day (Fig. 5e).

We subsequently analysed data from these experiments to determine the impact of acute Arch-inhibition on the circadian activity of VIP cells and their downstream targets (see Methods). This manipulation did not completely silence VIP cells, which continued to express daily variations in firing during Arch-inhibition, albeit with substantially diminished amplitude (Fig. 5c). As a consequence, the portions of the day when VIP cells were firing at a high rate (>50% of the spontaneous maximum) was severely truncated under these conditions (Fig. 5g). In line with these changes, optogenetic inhibition of SCN VIP cell activity produced a corresponding increase in the duration of the VIP^in^ cells’ active epoch (Fig. 5g). These data, therefore, support the view that inhibitory input from SCN VIP cells substantially influences the circadian firing patterns VIP^in^ cells.

**Fig. 6:**
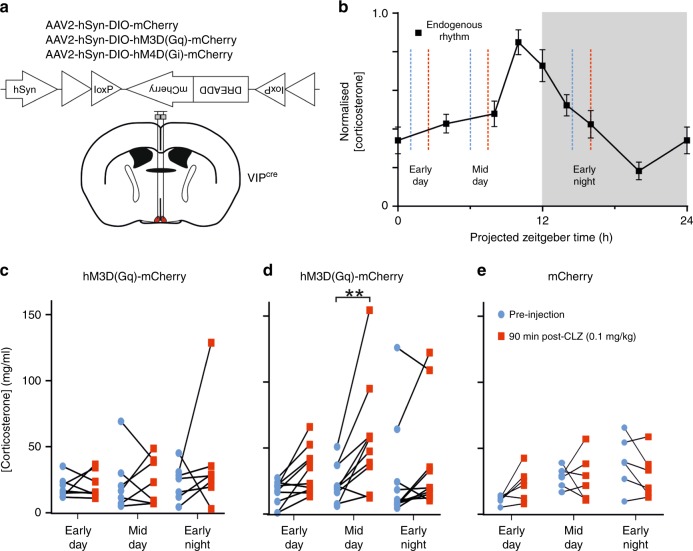
Chemogenetic inhibition of SCN VIP cells modulates circulating corticosterone concentrations. **a** Schematic showing the viral constructs delivered bilaterally to the SCN of *VIP*^*+/cre*^ mice. **b** Normalised daily changes in corticosterone concentration for wild type animals (*n* = 12; mean ± SEM) with schematic indication of epochs used for analysis of response to DREADD-based manipulation: tail bleed samples were taken immediately prior to injection of clozapine (CLZ; 0.1 mg/kg) and 90 min post-injection (blue and red dotted lines respectively) over three different time-points. Testing across each of the three indicated time-points was separated by at least 5 days. **c**–**e** Circulating corticosterone concentrations prior to- and following clozapine injection in Gq- (**c**; *n* = 7), Gi-DREADD (**d**; *n* = 10) and mCherry (**e**; *n* = 6) animals. Data were analysed by two-way RM ANOVA (**c**; CLZ:  F_1, 6_ = 0.66, *P* = 0.45; Epoch: F_2, 12_ = 1.07, *P* = 0.37; Epoch X CLZ: F_2, 12_ = 0.72, *P* = 0.51; **d**: CLZ: F_1, 9_ = 29.16, *P* = 0.0004; Epoch: F_2, 18_ = 1.31, *P* = 0.29; Epoch X CLZ: F_2, 18_ = 2.08, *P* = 0.15; **e**: CLZ: F_1, 5_ = 0.6, *P* = 0.47; Epoch: F_2, 10_ = 3.12, *P* = 0.08; Epoch X CLZ: F_2, 10_ = 2.18, *P* = 0.16). Sidak’s post-tests at individual epochs were performed on data form Gi-DREADD expressing animals (**d**; where ANOVA reported a significant main effect of CLZ). **P < 0.005.

We further found asymmetrical changes in circadian waveform of VIP^in^ cell firing. Specifically, analysis of both absolute and proportional changes in spike output revealed a phase-dependent modulation of VIP^in^ cell responses, with consistently robust increases in activity when Arch-stimulation coincided with the daily rise rather than decline in spontaneous firing (Fig. 6h, i). By contrast, Arch-mediated inhibition of SCN VIP cells did not display an equivalent phase-dependency (Fig. 5h, i). This indicates that the asymmetrical increases in VIP^in^ cell firing reflect circadian variation in the degree of spontaneous inhibitory input from SCN VIP cells and highlights a primary role for SCN cell group in controlling the daily rise in VIP^in^ cell firing.

### Impact of VIP cell output on physiology

Having established that circadian output from SCN VIP cells shapes rhythmic activity in downstream recipient neurons, we next investigated the physiological significance of this clock output pathway. Here, we employed a chemogenetic strategy to acutely modulate VIP cell output, by microinjecting viral constructs encoding Cre-dependent Gq- or Gi-coupled DREADDs or control vector into the SCN of *VIP*^*+/cre*^ mice (Fig. 6a). As expected^31,44^, this resulted in strong transfection of neurons within the ventral, VIP-cell rich, region of the SCN in *VIP*^*+/cre*^ mice but no transfection in *VIP*^*+/+*^ animals (Supplementary Fig 7a–c).

We then used this approach to examine the impact of VIP cell activity on circulating corticosterone, a major clock-controlled endocrine signal where a potential regulatory influence of SCN VIP neurons has previously been postulated^11^. To this end, we compared circulating corticosterone in virally transfected mice before and 90 min following injection of vehicle or a DREADD-selective^45^ dose of clozapine (CLZ; 0.1 mg/kg; see Methods). Based on our neurophysiological data and the endogenous diurnal profile of circulating corticosterone in mice (Supplementary Fig. 8), we performed these studies over three different epochs (Fig. 6c), where endogenous corticosterone levels were stable and sub-maximal but spontaneous VIP cell activity was high (mid-day) or low (early-day and early-night).

Vehicle administration did not significantly alter circulating corticosterone levels at any time-point for any of the experimental groups (Supplementary Fig. 8b–d). Similarly, in Gq-DREADD-expressing mice we did not find any significant effect of activating SCN VIP cells across any of the test epochs (Fig. 6c), nor did CLZ injection result in significant changes in circulating CORT in control vector expressing mice (Fig. 6e). By contrast, chemogenetic inhibition of VIP cells in Gi-DREADD-expressing animals significantly increased circulating corticosterone, with particularly robust effects at the mid-day epoch (Fig. 6d). Accordingly, the observed changes in circulating CORT (relative to pre-injection levels) in these Gi-DREADD-transfected animals were significantly larger than those for the vector control group (two-way mixed effects ANOVA; virus: F_1,14_ = 14.8, *P* = 0.002; Epoch: F_2,28_ = 2.05, *P* = 0.15; Epoch X virus: F_2,16_ = 1.49, *P* = 0.24; Sidak’s post-test at mid-day timepoint, *P* = 0.01). This mid-day time-point, where greatest effects were observed, corresponds to the normal peak in spontaneous SCN VIP cell activity. These data, therefore, suggest that the endogenous activity of SCN VIP cells is especially important for supressing day-time corticosterone release, ensuring that the daily rise is timed to occur just-prior to activity onset.

To further validate these findings, we also confirmed that virally delivered Gi- and Gq-DREADDs effectively modulate the activity of SCN VIP cells. Gi-DREADD-mediated inhibition of SCN VIP neurons was previously shown to prevent neuronal activation following nocturnal light pulses^36^. Accordingly, we here found in ex vivo recordings from Gi-DREADD-transfected VIP-ChR2 mice (where the DREADD-mCherry and ChR2-EYFP signals strongly co-localise; Supplementary Fig. 7d) that CLZ application during the projected day significantly inhibited the spontaneous firing of optogenetically identified SCN VIP cells (Supplementary Fig. 7e, f). Moreover, that effect was associated with significant increases in the firing of VIP^in^ cells located outside the SCN (Supplementary Fig. 7e, f). Similarly, we found that in vivo activation of chemogenetic constructs in Gq-DREADD transfected *VIP*^*+/cre*^ mice at ZT14.5 was sufficient to significantly elevate c-Fos expression in the SCN (Supplementary Fig. 7g, h). These data, therefore, confirm that our DREADD-based strategy effectively modulates SCN VIP cell output and highlights an important role for this pathways in regulating endogenous rhythms of circulating CORT.

We next used the same approaches to investigate the contribution of SCN VIP cells to regulating other key physiological outputs under clock control, namely heart rate and locomotor activity. Thus, a subset of Gq- (*n* = 5), Gi-DREADD (*n* = 5) and control vector (*n* = 6) transfected *VIP*^*+/cre*^ mice were implanted with radiotelemetry remotes, allowing untethered, home cage, monitoring of heart rate and activity. The impact of chemogenetic activation or inhibition of SCN VIP cells was then investigated at equivalent time-points to those used for assessment of circulating corticosterone.

**Fig. 7:**
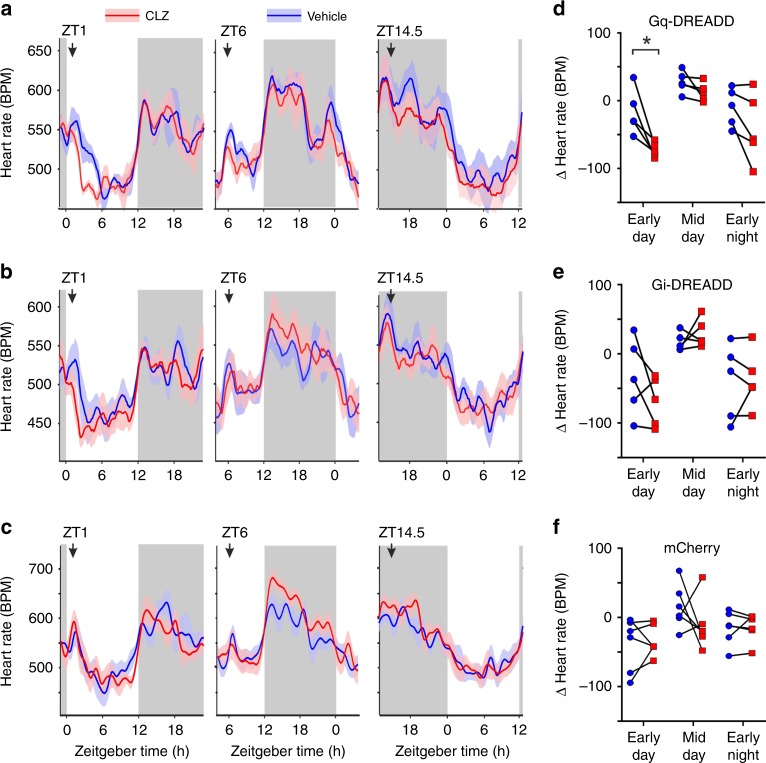
Chemogenetic excitation of SCN VIP cells modulates heart rate during the early day. **a**–**c** Heart rate following vehicle or clozapine (CLZ; 0.1 mg/kg i.p.) injections at varying portions of the daily cycle in animals expressing Gq- (**a**), Gi-DREADD (**b**) or mCherry (**c**) in SCN VIP cells. Arrows above traces indicate timing of injections; grey shaded areas represent time of lights off. **d**–**f** Change in heart rate 1–5 h post vehicle or clozapine injection (relative to mean values in the 2 h epoch preceding injection) for Gq- (**d**), Gi-DREADD (**e**) and mCherry (**f**) expressing mice. Data were analysed by two-way RM-ANOVA (**d**: CLZ: F_1, 4_ = 891.3, *P* < 0.0001; Epoch: F_2, 8_ = 16.57, *P* = 0.0014; Epoch X CLZ: F_2, 8_ = 1.84, *P* = 0.22; **e**: CLZ- F_1, 4_ = 0.52, *P* = 0.51; Epoch: F_2, 8_ = 6.76, *P* = 0.02; Epoch X CLZ: F_2, 8_ = 2.06, *P* = 0.19; **f**: CLZ: F_1, 5_ = 0.52, *P* = 0.50; Epoch: F_2, 10_ = 3.48, *P* = 0.07; Epoch X CLZ: F_2, 10_ = 1.28, *P* = 0.32). Sidak’s post-tests at individual epochs were performed on data form Gq-DREADD expressing animals (**c**; where ANOVA reported a significant main effect of CLZ). *P < 0.05.

Inhibition of SCN VIP cells did not significantly alter heart rate (Fig. 7b, d) or activity levels (Supplementary Fig. 9b, e) across any of the analysed time-points. Similarly CLZ injection did not significantly impact heart rate or activity in control vector-transfected *VIP*^*+/cre*^ mice (Fig. 7c, f; Supplementary Fig. 9c, f). By contrast, Gq-DREADD-driven activation of SCN VIP cells significantly reduced heart rate relative to matched vehicle injections, with most reliable effects observed during the early-day a time-course consistent with previously reported DREADD effects^46^ (Fig. 7a, d). Importantly, this effect on heart rate was not due to suppression of activity (Supplementary Fig. 7a). Hence, while analysis of the simultaneously acquired activity data did reveal a significant effect of CLZ in this group, changes observed in activity levels specifically during this early-day epoch were essentially identical for vehicle and CLZ (Supplementary Fig. 9d, Sidak’s post-test: *P* = 0.47). We did, however, find a modest suppression of activity following activation of SCN VIP cells during the early night (Sidak’s post-test *P* = 0.04), consistent with a prior report that optogenetic activation of VIP cells during this time-point suppresses locomotor activity^30^. Accordingly, while our data support this view that VIP cell output can acutely regulate locomotor activity they also indicate a specific role in regulating daily rhythms in cardiac output that is independent of this effect.

## Discussion

Traditionally, VIP cells have been considered critical for internal SCN timekeeping, but less so for the downstream communication of clock signals^18–21,47,48^. Here we show that, in fact, individual VIP cells, in acute adult slice preparations, exhibit robust and tightly coordinated daily variation in spontaneous firing that shapes cellular rhythms in electrophysiological activity across major SCN target nuclei. We further show that acute modulation of VIP cell activity impacts daily rhythms in heart rate and circulating glucocorticoids in a temporally restricted manner, indicating that endogenous VIP cell output regulates key aspects of clock-controlled physiology.

In line with our data, recent studies indicate that VIP cells also sustain population-level circadian rhythms in *Per2* & *Cry1* transcription, intracellular Ca^2+^ and possibly membrane voltage^31–33^. Similarly, cultured neonatal SCN VIP neurons typically exhibit robust circadian variation in spontaneous firing rate^30^, suggesting electrophysiological rhythms are generated by VIP neurons in a cell-intrinsic manner. Accordingly, we here find that the circadian activity profiles for individual VIP cells in adult SCN slice preparations are similar to those of other SCN neurons. As a population, however, VIP cell output rhythms are more closely synchronised than for non-VIP cells. This arrangement supports previous suggestions that the SCN contains differentially phased subpopulations of cells with distinct functional roles^11,12^ and likely contributes to the increased population level daytime firing rates reported previously for VIP vs. other SCN neuron types^35^.

Consistent with the view that this coordinated VIP cell population activity plays a specific role in conveying temporal information to cells outside the SCN, we find a subset of neurons across known anatomical target sites that receive inhibitory input from VIP cells. The anatomical distribution of these VIP^in^ cells matches SCN VIP cell projections as reported by ChR2-EYFP (present study) and immunohistochemically^8^, with such neurons clustering at the level of the SPZ and dorsal cap of the PVN. We further show that VIP^in^ cells exhibit circadian variations in firing with a nadir coinciding with the endogenous peak in SCN VIP cell activity and confirm a causative link between these observations by demonstrating that appropriately timed manipulation of VIP cell firing (by ChR2 stimulation or Arch-mediated inhibition) substantially modulates ongoing VIP^in^ cell activity.

Interestingly, we find that the primary mechanism by which VIP cells influence these target neurons is via GABA rather than VIP release. This observation is consistent with anatomical data indicating that VIP receptors are almost entirely absent from the SPZ, PVN and ventral thalamus in mice^42,43^. By contrast, VPAC_2_ receptors have been detected in the rat PVN^49^, which may explain previous observations that exogenous VIP^50–52^ can modulate corticosterone secretion in that species. While these pharmacological studies are not necessarily indicative of the physiological roles of endogenous VIP signalling, it seems possible that the relative contributions of GABA and VIP signalling to SCN VIP cell output may differ between species.

Of note, we also find that the timing of peak firing among VIP^in^ cells is substantially more variable than that of VIP cells themselves, implying that these daily variations in firing do not entirely reflect a passive response to circadian rhythms in inhibitory GABAergic input from SCN VIP cells. Rhythmic expression of core clock genes has been observed in those locations where we find VIP^in^ cells^53–55^, thus local clocks might also (directly or indirectly) contribute to the circadian variation observed in their activity. Similarly, SCN cells other than those that express VIP also send projections to SPZ, PVN and ventral thalamus^8–10^ and could, in principle, also influence the firing of VIP^in^ cells in some way. Naturally, in vivo, additional rhythmic factors not present in our slice preparation could also influence the temporal profile of VIP^in^ cell activity, although the population rhythms we observe correspond well with those obtained in recordings from a key VIP cell target region (SPZ) in freely-behaving animals^56^.

The above not-withstanding, it is abundantly clear that the circadian timing information provided by SCN VIP cells is not only a significant influence on the electrophysiological activity of VIP^in^ cells but, also, shapes daily variations in crucial features of whole-animal physiology. Accordingly, we find that chemogenetic inhibition of SCN VIP cells during their mid-day peak results in marked increase in circulating corticosterone; a major systemic regulator of cell physiology and source of circadian timing information for peripheral tissue clocks^57^. Specifically, our data indicate that endogenous circadian variation in VIP cell output shapes the daily rhythm in glucocorticoid secretion by suppressing release during the mid-late day, ensuring that the daily rise in circulating corticosterone is timed to occur just before the active phase.

Previous attempts to understand the neural circuits underlying circadian control of corticosterone in rats suggest that AVP release from the SCN provides a similar inhibitory control during earlier portions of the day^58,59^. This is believed to reflect a stimulatory of effect of AVP on inhibitory interneuorns that innervate corticotrophin-releasing hormone (CRH) cells in the PVN^11^. Such a mechanism potentially accounts for our observation that chemogenetic activation of SCN VIP cells during the early day (when endogenous activity is low) does not noticeably suppress levels of circulating corticosterone. Similarly, the lack of effect following activation of SCN VIP cells during the early night presumably reflects the fact that the nocturnal decline in circulating corticosterone is primarily driven by its own feedback-inhibition of hypothalamic-pituitary-adrenal axis activity^15^.

By contrast to the above, our observation that inhibition of SCN VIP cell activity drives increases in circulating corticosterone during the mid-late day could most simply be interpreted as reflecting a direct inhibitory control on CRH neuronal activity. However, both VIP cell efferents^8^ and VIP^in^ cells are sparse within the PVN regions that containing CRH neurons^43^. A polysynaptic connection between SCN VIP cells and the PVN CRH population is certainly possible (involving interneurons in the SCN and/or other SCN targets) but we do not find evidence for a major output of this nature in our electrophysiological studies employing opto- and/or chemogenetic manipulations, with very few neurons displaying the hallmarks of polysynaptic activation.

In sum, we think it more likely that chemogenetic inhibition of SCN VIP cells impacts circulating corticosterone via a mechanism independent of the CRH pathway. Accordingly, the dorsal cap of the PVN (which our data highlight as a key target of SCN VIP cell output) contains numerous spinally projecting neurons^60,61^. Since SCN-dependent changes in the activity of pre-autonomic neurons in the PVN provides an additional source of control over the daily rise in circulating corticosterone (by adjusting adrenal cortex sensitivity to ACTH^15,62,63^), VIP cell input to such spinally projecting neurons appears the most likely candidate for the effects seen here. While future studies will be required to definitively resolve this issue, the previous observation that SCN VIP cells innervate PVN neurons that are multisynaptically connected to the adrenal^63^ certainly supports the mechanism suggested above.

We also show that VIP cell stimulation produces a prolonged decrease in heart rate with especially robust effects during early-day. These changes in heart rate are not associated with any overt effects on behavioural activity, although we do find evidence that activation of SCN VIP cells can supress behavioural activity during the early-night (as reported previously for optogenetic stimulation^30^). These data, therefore, suggest a specific role for VIP cells in regulating daily variations in cardiac function. Since chemogenetic inhibition of SCN VIP cells during their mid-day peak does not produce correspondingly large increases in heart rate, we infer that the ability of endogenous VIP cell firing to influence heart rate is restricted under our experimental conditions. This presumably reflects other ongoing rhythmic process that impact heart rate throughout the diurnal cycle, including gross changes in activity, arousal state and autonomic tone as well as potentially circadian clock mechanisms located in the heart and/or circuity downstream of the SCN^53–55,64^.

Regarding the circuitry responsible for effects of VIP cell activity on heart rate, as above, the dorsal cap of the PVN where we find VIP^in^ cells contains spinally projecting neurons^60,61^, as well as cells projecting to the rostral ventrolateral medulla^65^. Either/both populations of neurons could contribute to the effects on heart rate observed here^66^. Certainly our analyses of electrophysiological responses imply that direct effects of VIP cell output on this PVN cell group provide the most likely (and simplest) potential origin for such effects, as discussed above for corticosterone. Future studies would be required to definitively rule out alternative, less direct, actions however (e.g., multisynaptic pathways involving the SPZ and DMH).

Regardless of the precise neural circuits involved, our findings establish VIP-expressing SCN neurons as a major source of circadian timing information for downstream neural circuits implicated in neuroendocrine and autonomic control. In line with other emerging data indicating that the SCN employs specifically timed neural outputs to control distinct aspects of physiology and behaviour^16,67^, these findings provide vital new insight into the mechanisms by which the central clock aligns whole body function to the environment.

## Methods

### Animals

All animals were used in accordance with the Animals, Scientific Procedures, Act of 1986 (UK) and received both institutional ethics committee and UK Home Office approval. Experimental animals were generated by crossing VIP-IRES-Cre mice (JAX #010908)^68^ and animals bearing Cre-dependent channelrhodopsin2 (ChR2)-EYFP (Ai32; JAX #0102569)^69^ or Archaerhodopsin 3-EYFP (Ai40D; JAX #021188) constructs, as appropriate. Mice were housed in 12:12-h light/dark cycles in a temperature controlled environment (22 °C). Zeitgeber time (ZT) 0 was designated as time of lights-on and ZT12 as lights-off. Food and water were provided ad libitum.

### Ex vivo electrophysiological recordings

Electrophysiological experiments were performed on male and female mice (age 41–164 days), that were heterozygous for VIP-IRES-Cre and either Ai32 (*VIP*^*+/cre*^*; Ai32*^*+/*−^, termed VIP-ChR2; experiments presented in Figs. 1–4 and Supplementary Figs. 1–7) or Ai40 (*VIP*^*+/cre*^*; Ai40*^*+/*−^, termed VIP-Arch; experiments presented in Fig. 5). Where relevant, control data was obtained from *VIP*^*+/+*^*; Ai32*^*+/*−^ animals (Supplementary Fig 4).

Mice were removed from the home cage at ZT0-1 or ZT11-12 and culled via cervical dislocation, followed by decapitation and brain removal. Coronal slices (350 μm thickness) containing the mid-rostrocaudal extent of the SCN (where VIP cells are concentrated^8^) and the PVN were prepared using a 7000 smz-2 vibrating microtome (Campden Instrument, UK). Slicing was performed in an ice cold sucrose slicing solution (4 °C; sucrose (189 mM); D-glucose (10 mM); NaHCO_3_ (26 mM); KCl (3 mM); MgSO_4_ (5 mM); CaCl_2_ (0.1 mM); NaH2PO4 (1.25 mM)), oxygenated with 95% O_2_/5% CO_2_. Slices were subsequently placed in aCSF solution (NaCl (124 mM); KCl (3 mM); NaHCO_3_ (24 mM); NaH_2_PO_4_ (1.25 mM); MgSO_4_ (1 mM); glucose (10 mM); CaCl_2_ (2 mM), 0.0001% gentamicin (Sigma-Aldrich, UK)), oxygenated with 95% O_2_/5% CO_2_ and left to rest at room temperature for ~20 min. Slices were further equilibrated within the recording setups for 1–2 h prior to the start of electrophysiological data collection.

For multielectrode array recordings, procedures were similar to those described by Hanna et al.^70^. Slices were aligned on 6 × 10 perforated multielectrode arrays (pMEAs; Multichannel Systems, MCS, Germany; Fig. 1), such that recording sites spanned either the SCN and SPZ, or the PVN, SPZ and ventral thalamus. Slice position was visualised under white light trans-illumination using a GXCAM-1.3 camera attached to a dissecting microscope (GX optical, UK) and snapshots were taken to verify placement over electrode sites. Whilst in the recording chamber both slice surfaces were constantly perfused with warm oxygenated aCSF (33 °C ± 1 °C) at a rate of ~3 ml a minute. Consistent flow was driven by a constant vacuum pump (MCS gmbH, Germany) removing waste aCSF, and a peristaltic pump (120S Watson-Marlow, Falmouth, UK; flow rate ~3 ml/min) delivering fresh oxygenated aCSF. Slice position was maintained using a weighted harp (ALA Scientific Instruments, NY, USA), alongside the suction caused by the flow of aCSF through the perforated MEA array. Neural activity was then acquired continuously over >26 h using MC_Rack software via a USB-ME64 system and MEA1060UP-BC amplifier (MCS GmbH, Germany). Signals were sampled at 50 kHz and high pass filtered at 200 Hz (Second order Butterworth). Spikes crossing a threshold (normally set at −16.5 µV) were then extracted as timestamped waveforms (1.5 ms duration) and single unit activity was isolated offline by principle components based spike sorting using Offline Sorter (V3; Plexon, TX, USA) and NeuroExplorer (V5; Nex Technologies).

For ex vivo recordings using penetrating multisite electrodes, coronal brain sections (prepared as described above) were transferred to a heated submerged slice chamber (BSC-1; Digitimer Ltd., Welwyn Garden City, UK) and held down by a weighted harp (ALA Scientific Instruments). Bath temperature was maintained at ~33 °C and fresh, pre-warmed and oxygenated aCSF was supplied by peristaltic pump (120S Watson-Marlow; flow rate ~1.5 ml/min). Silicon 32-channel electrodes (Buszaki 32L, NeuroNexus, MI, USA), each consisting of 4 shanks with 8 closely spaced electrodes (see Fig. 4), were then positioned within the brain slice (with the aid of a dissecting microscope) such that recordings sites either spanned the SPZ, PVN and ventral thalamus or (for Gi-DREADD validation in Supplementary Fig 6d–f) the SCN, SPZ and PVN. Neural signals were acquired by a SmartBox system (Neuronexus) at 20 KHz with spike extraction and single unit isolation performed on virtual tetrode waveforms as described in Howarth et al.^71^ using custom Matlab (Mathworks, MA, USA) scripts and Offline sorter software.

### Pharmacological manipulation

At the end of all experiments slices received 5 min bath applications of N-methyl-D-aspartic acid (NMDA, 20 μM) to confirm viability, followed by tetrodotoxin citrate (TTX, 1 μM), to confirm that acquired signals exclusively reflected Na+-dependent action potentials. Where relevant, slices also received one or more of the following compounds, singly or in combination: VIP (100 nM), the VIP receptor antagonist [D-p-Cl-Phe6,Leu17]-VIP (1 µM), (+)-bicuculline (BIC, 20 µM) to block GABA_A_ receptors and/or D-(-)-2-amino-5-phosphonopentanoic acid (D-AP5, 50 µM) and 6-cyano-7-nitroquinoxaline-2,3-dione disodium (CNQX, 20 µM) to block ionotropic glutamate receptors. For Gi-DREADD validation experiments, slices received clozapine (CLZ, 100 nM). All compounds were obtained from Sigma-Aldrich (Poole, UK) or Tocris (Abingdon, UK). Drugs were stored as 1000X stock solutions at −20 °C (In ddH_2_O or, in the case of BIC and CLZ, DMSO) and were diluted to working concentrations in pre-warmed, oxygenated, aCSF just prior to use.

### Optogenetic manipulation

For all experiments, an optical fibre (200 µm core; 0.66NA; Plexon) was positioned directly above the SCN (~100–200 µm above slice surface). For studies employing ChR2, illumination was provided by a PlexBright tabletop 460 nm module (Plexon), providing ~792 mW/m^2^ output at the fibre tip. For long-term pMEA experiments, we applied ‘ramped’ stimuli (consisting of a sequence of 60–140 µs flashes for a total of 3 ms illumination over a 9 ms epoch) at a rate of 1/min for the duration of the recording (>26 h). For acute experiments using penetrating electrodes, square wave 10 ms pulses were applied at varying interstimulus intervals; either as single pulses (0.5 Hz), pairs with ISIs spanning 0.5–40 Hz (repeating interleaved sequence at 2 s/pair) or stimulus trains with rates from 0.5–40 Hz (repeating 4 s interleaved blocks separated by 5 s of no stimulation). For long-term pMEA experiments employing Arch, illumination was provided by a fibre coupled 560 nm LED module (Thorlabs, Ely, UK), providing ~140 mW/mm^2^ at the fibre tip. Here light was provided as 10 s steps every 5 min for the duration of the recording.

### Data analysis

Analysis of long-term pMEA recordings was performed using Matlab routines, as described previously^70^. Isolated neuron firing rates were considered to exhibit circadian variation when better fit by a sinusoidal function (constrained to a periodicity of 20–28 h) than a first order polynomial. For rhythmic channels, the projected ZT of peak firing was then determined from a 60 s binned time-series (smoothed with a 2 h boxcar filter). Peak width and peak trough amplitude were determined from this smoothed time-series, with the former representing the duration over which cell firing was >50% of the peak within a single 24 h cycle. For comparison of cell phasing between populations, Rayleigh analysis was used to determine circular median and data were subsequently adjusted to a relative phase such that, within each dataset, each observation was no more than 12 h displaced from the population median. This linearisation procedure allowed for conventional tests to assess differences in variance across the population (Browne–Forsythe’s test) and the corresponding mean phase. Since we identified variable numbers of cells corresponding to the populations of interest from each slice recording, for the latter (and all other group-wise comparisons between derived measures of individual cell firing activity) we used a multilevel mixed-effects linear model that included the slice that each cell was recorded from as a 2nd-level random effect (SPSS, IBM, NY, USA).

Acute changes in neural activity evoked by optogenetic stimuli were classified as excitatory or inhibitory when the average spike counts across multiple stimulus repeats (typically substantially >100) respectively exceeded the upper or lower bounds of the 99% confidence limits for prestimulus spike counts (NeuroExplorer v4; Nex technologies, CO, USA). Latency was determined from the first post-stimulus bin to exceed this boundary. Where cells exhibited fast excitatory responses to ChR2 excitation (peak response detected within 10 ms of the light stimulus) they were considered VIP-expressing cells. Cells matching these criteria (exclusively located in the SCN) were easily distinguishable from rarely encountered cells in SCN target regions showing weaker/more sluggish excitatory responses (slow-activation). To asses synaptic contributions to optogenetically driven responses, within each relevant experimental block (25 min epochs prior to and during drug application) we calculated the mean stimulus-driven changes in spike firing within an appropriate temporal window (25 ms for single pulse stimuli, 5s for trains, individually tailored for paired stimuli based on the duration over which significant changes in spike rate were observed). Responses were subsequently compared using a multilevel mixed-effects linear model that included the slice that each cell was recorded from as a 2nd-level random effect (as above) and stimulation frequency and/or drug as repeated fixed factors as required. Planned post-hoc comparisons were then applied as appropriate where significant main effects of the fixed factors were detected.

For comparison of observed vs. predicted responses to paired optogenetic stimulation (Supplementary Fig. 6c), we first calculated post-stimulus spike count histograms for each cell from 0 ms to 500 ms following low frequency stimulation (LFS, 0.5 Hz) of SCN VIP cells. Predicted response profiles for paired stimulation were then calculated as a simple linear sum of the initial response histogram (extracted above) and an appropriately time-shifted version of the same (with the caveat that the resulting spike counts firing rates were constrained to have a minimal possible value of zero). We then calculated the mean change in firing rate for each cell between response onset and offset (first and last bins were firing rates were significantly below baseline) for both predicted and measured response to LFS and stimulus pairs and normalised the resulting data series to have maximal value of one. Group data processed in this way were then analysed by multilevel mixed-effects linear modelling as above.

For analysis of data from VIP-Arch slices (Fig. 5), cell classification and analysis procedures were essentially identical to those use for VIP-ChR2 with minor modifications. Specifically, to compare circadian profiles in the presence/absence of Arch mediated-inhibition of SCN VIP cells, we first took the raw spike counts over time, extracted from these epochs with and without illumination, and then used spline based interpolation to produce the corresponding time-series of mean firing rate (spikes/s) with 60s resolution, before subsequent processing as above. In addition, we specifically compared firing rates in the presence and absence of Arch mediated-inhibition across specific portions of the circadian profile for each cell (trough, peak, rising and falling; defined respectively by the lower and upper quartiles of the cells firing profile and the corresponding intervening regions). For these comparisons, we assessed both absolute and relative changes in firing rate. In the case of the latter we calculated a standard modulation index of the form (Arch−spontaneous)/(Arch+spontaneous), The resulting measure was therefore bounded between −1 and 1 (for Arch-mediated decreases and increases in firing rate respectively).

Estimates of the anatomical localisation of recorded neurons were obtained from snapshot images of the slice/recording electrode placements, with recording electrodes (and units detected therein) being assigned as located within or outside the SCN based on the observable anatomical boundaries of the nucleus for each slice, evident in the white light trans-illuminated images. Prior analysis^70^ indicates that the actual positions of recorded neurons are likely to lie within ±17 µm of the recording site centres. Coupled with the fact that the precise anatomical boundaries between the extra-SCN regions studied here were not always as unambiguously identifiable in the slice images as for the SCN (in particular the PVN/SPZ border), we did not categorise these extra-SCN neurons to specific target structures. Instead, we estimated anatomical positions based the known electrode array geometries relative to fixed landmarks within the slice; specifically the distance between each recording site and the intersection of best fit lines through the 3rd ventricle and the ventral border of the SCN/optic tract. We then present these data as either binned proportions of identified cells across the dorsal-ventral axis of the slice (Figs. 2b and 4a) or as estimated positions (Supplementary Fig 5b) mapped onto a standardised anatomical template derived from the Mouse Brain Atlas^72^.

### Viral injections

Viral injections were performed on male and female mice (49–157 days old). Experiments were primarily performed in *VIP*^*+/cre*^ individuals (Figs. 6 and 7 and Supplementary Figs. 8 and 9). For additional validation of viral specificity (Supplementary Fig. 7b, c), injections were performed in *VIP*^*+/+*^ animals. For electrophysiological validation of the Gi-DREADD approach (Supplementary Fig. 7d–f) mice were heterozygous for both VIP-IRES-Cre and Ai32 (i.e. *VIP*^*+/cre*^; Ai32^+/−^).

Mice were anaesthetised using 1% isoflurane in O2, and placed into a stereotaxic frame following which the skull was exposed. Craniotomy and bilateral injection of virus was completed using a motorised injector under computer control (Drill and 473 Microinjection Robot: Neurostar, Tϋbingen, Germany). Subsequently, 69 nl of viral vector encoding an activating or inhibiting DREADD or control construct (AAV2-hSyn-DIO-hM3D(Gq)-mCherry, AAV2-hSyn-DIO-hM4D(Gi)-mCherry or AAV2-hSyn-DIO-mCherry respectively; Addgene, MA, USA) was delivered at 25 nl/s. Micropipettes were left in place for 5 min following injection, and were then retracted in 100 µm/5s increments until above the SCN.

### Corticosterone measurements

Tail vein blood samples (5 µl) were collected, following needle puncture, into heparinised capillary tubes (Drummond Microcaps, 5 μl). Samples were dispensed into 20 µl of PBS and placed on ice before centrifuging (VWR Micro Star 17r; 10,000× for 10 min 4 °C), with the resulting supernatant stored at −80° until analysis.

To assess diurnal variation in cortisol level, samples were collected at 9 time-points across the day-night cycle. Collections completed during the dark phase of the animals were performed under dim red light and animals were allowed sufficient time to recover between sample collections to allow tail healing and stress-induced corticosterone dissipation (minimum 3 days between sampling). Tail blood sampling for DREADD expressing animals was performed at defined epochs across the diurnal cycle (early day, mid-day and early night). Blood was initially sampled at ZT1, 6 or 14.5, immediately followed by injection of a DREADD-selective^45^ dose of clozapine (0.1 mg/kg i.p.; Tocris) or vehicle (0.01% DMSO in saline). This dose of clozapine has previously been shown to produce comparable effects to those commonly used for the original DREADD activator (10 mg/kg), which does not cross the blood brain barrier and therefore requires metabolic conversion to clozapine in order to act centrally^45^. Moreover, at 0.1 mg/kg, the clozapine dose used here is ~10-fold lower than the threshold for non-DREADD mediated effects in other systems^45,73^.

Ninety min post injection a second blood sample was collected (a timeframe chosen to encompass the expected peak in DREADD effects). Testing at each epoch was separated by at least 5-days to avoid any lingering effect of the prior manipulations.

In all cases, serum samples were subsequently analysed using pre-coated ELISA plates (ABCAM, ab108821; Cambridge, UK). Concentration changes were assessed using two-way RM ANOVA with Sidak’s multiple comparisons (GraphPad Prism, CA, USA).

### Radiotelemetry

Mice were anaesthetised using 1% isoflurane in O_2_. Once deeply anaesthetised, an incision was made into the peritoneal cavity and ETA-F10 radio telemetry devices (Data Sciences International; MN, USA) inserted. The muscle lining was sutured and the electrodes of the ECG telemetry remote were routed subcutaneously and sutured to lie on either side of the heart. Once electrodes were in position the wound was closed. Animals were administered analgesia (Buprenorphine 0.01 mg/kg) and left to recover for one week before the start of experiments. During the experiment, mice were singly housed (with ad libitum food) under a 12:12 h light-dark cycle. ECG/activity signals were acquired by a receiver pads placed under the mouse’s home cage (RPC-1; Data sciences international). Activity counts and heart rate (bpm) were acquired in 5 min intervals for the duration of the experiment. For assessment of DREADD-based manipulation of SCN VIP cell activity commenced, animals received vehicle injection (0.01% DMSO in saline, i.p.) at one of three time-points (ZT 1, 6 or 14.5) and subsequently a DREADD-selective clozapine injection (0.1 mg/kg, i.p) at an equivalent timepoint the following day. Initial testing was preceded by a 3-day baseline recording epoch and at least five days drug free days were left between testing at each timepoint.

For visualisation of responses to chemogenetic manipulations, data (5 min bins) were smoothed with a Gaussian function (σ = 30 min). For subsequent analysis, we extracted the change in HR or activity 1–5 h post i.p. injection, relative to the mean value in the preceding 2 h epoch for each animal. This time range was chose to avoid acute changes in heart rate/activity induced by the injection and to encompass that over which DREADD-based responses were reported previously^45,46^. The resulting data were then analysed by two-way RM ANOVA with Sidak’s multiple comparisons (GraphPad Prism).

### Immunohistochemistry

At the end of experimental procedures, virally injected mice were deeply anaesthetised with urethane (10%; i.p. injection) and transcardially perfused with 4% paraformaldehyde. Brains were removed and post-fixed overnight in 4% paraformaldehyde, before being dehydrated in 30% sucrose. Sections were then cut at 50 μm on a freezing sledge microtome (8000 Sledge; Bright Instruments, UK) and blocked for 3 hs (10% Donkey serum, Sigma Aldrich; 0.2%PBS-T) at room temperature. To confirm viral expression, the intrinsic mCherry tag was amplified by incubation with primary antibody (rabbit anti-ds red; 1:2500; TaKaRa Bio, CA, USA; 2.5% donkey serum, 0.2% PBS-T) overnight at 4 °C. Following a 30 min wash (0.2%PBS-T), sections were incubated with secondary antibody (Alexa 546 donkey anti rabbit; 1:1000; Thermo Fisher Scientific; 2.5% donkey serum, 0.2%PBS-T) for 4 h at room temperature. Sections were then mounted using vectashield hardset with DAPI (Vector Labs, H-1500).

A subset of Gq transfected animals underwent concurrent staining for c-Fos activation. Clozapine (0.1 mg/kg, i.p) was delivered 90 min prior to transcardial perfusion, which occurred at ZT16. Protocol was as above, with additional primary antibody (chicken anti c-Fos; 1:200; Sigma Aldrich), and additional secondary antibody (Alexa 488 donkey anti chicken; 1:500; Thermo Fisher Scientific).

For visualisation of VIP cell terminal fields, *VIP*^*+/cre*^*; Ai32*^*+/*−^ mice were transcardially perfused, sectioned and processed as above, substituting the primary (chicken anti-GFP; 1:10,000; Abcam, Cambridge, MA, USA), and secondary antibodies (Alexa 488 donkey anti chicken; 1:500; Thermo Fisher Scientific) to allow visualisation of the intrinsic EYFP tag expressed by VIP cells in these animals. For DREADD virus expression (Supplementary Fig. 7d–f) these antibodies were used concurrently with those above for mCherry visualisation.

Images were collected on a Zeiss Axioimager.D2 upright microscope using a 10×/0.5 EC Plan-neofluor objective and captured using a Coolsnap HQ2 camera (Photometrics; AZ, USA) through Micromanager software v1.4.23. Images were then processed and analysed using Fiji ImageJ.

### Reporting summary

Further information on research design is available in the Nature Research Reporting Summary linked to this article.

## Supplementary information

Supplementary Information

Reporting Summary

## Data Availability

Materials, algorithms and data generated in these studies are available from the authors upon reasonable request.
