# Supplementary material for: Output from VIP cells of the mammalian central clock regulates daily physiological rhythms
Source: Nat Commun. 2020 Mar 19;11:1453. doi: 10.1038/s41467-020-15277-x (PMC7081308; doi:10.1038/s41467-020-15277-x)
Supplement: Supplementary file 1 — Supplementary Information [file 41467_2020_15277_MOESM1_ESM.pdf]

Output from VIP cells of the mammalian central clock regulates daily physiological rhythms

Paul et al.

Supplementary information

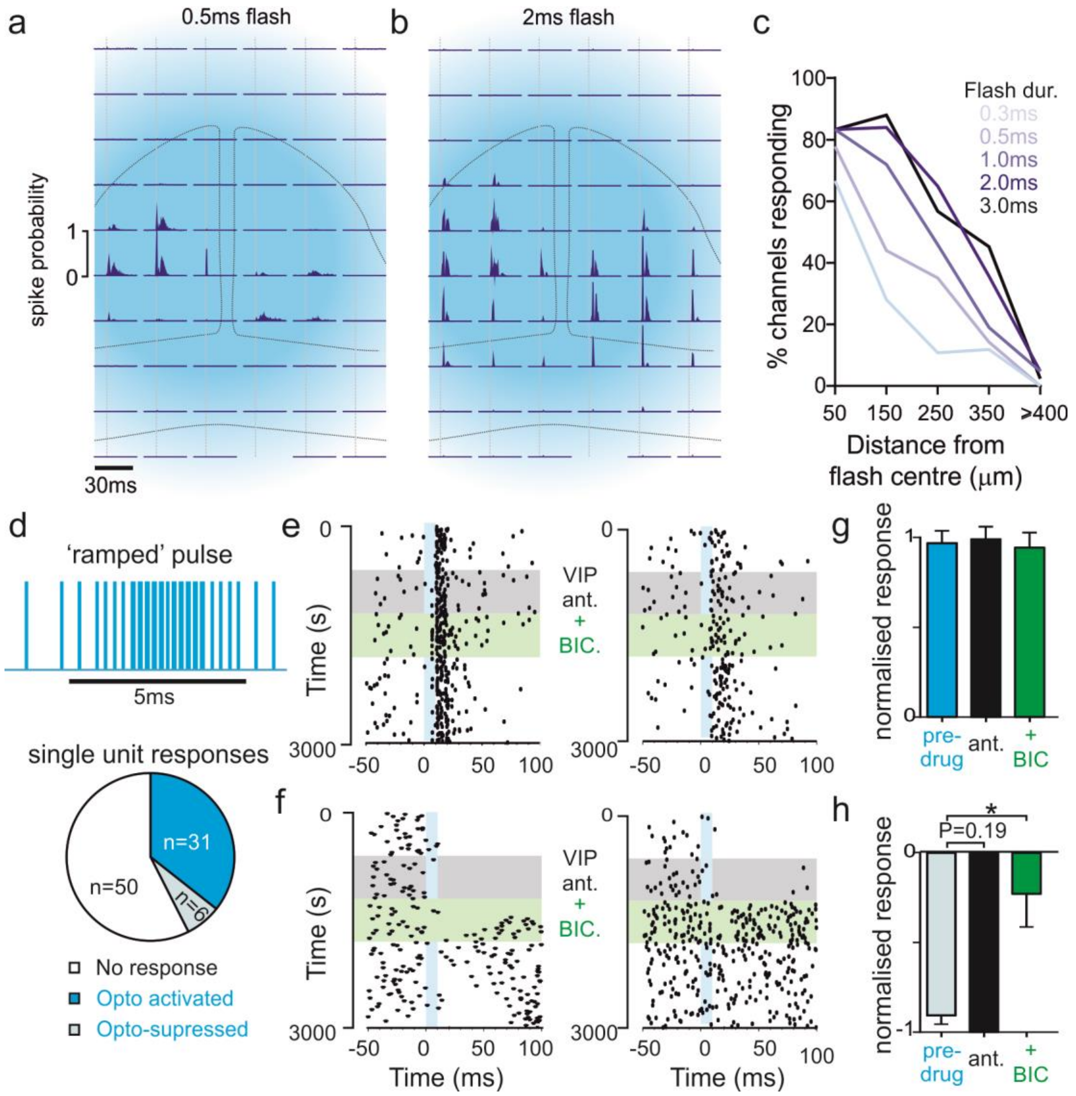

Supplementary Figure 1. Optogenetic identification of VIP-expressing neurons in the suprachiasmatic nucleus. (**a**&**b**) Multiunit spike probability (from 288 trials) at each electrode site of the 59-site array following 0.5 (**c**) and 2ms (**d**) light flashes via the optical fibre, from slice shown in Fig 1b**.** Overlays illustrate illuminated region and projected boundaries of the SCN and optic tract. (**c**) Fraction of channels responding to optogenetic stimulation as a function flash duration and the distance of each channel from the centre of stimulus (from n=211 active sites across 4 slices). (**d**) Temporal profile of ramped pulse stimuli used for single unit isolation (top) and fraction of isolated neurons activated or supressed by the stimulus (from 3 slices). (**e**&**f**) Ramped pulse-evoked spike raster plots showing representative examples of individual activated (**e**) and supressed (**f**) cells, before and after application of a VIP receptor antagonist ([D-p-Cl-Phe^{6},Leu^{17}]-VIP; 1µm, grey shading) followed by co-application with (+)-bicuculline (BIC; 20µm, green shading). Pulse timing shown as vertical blue shading. (**g**&**h**) Normalised mean±SEM responses of activated (**g**) and supressed (**h**) cells before and during application of VIP antagonist and co-application with BIC. Data analysed by multilevel mixed effects linear model (**g**: $F_{2,57.53}$=0.04, P=0.96; **h**: $F_{2,5.19}$= 10.65; P=0.02) with Dunnett's post-test applied to data from supressed cells (**h**), where a significant effect was detected; *=P<0.01.

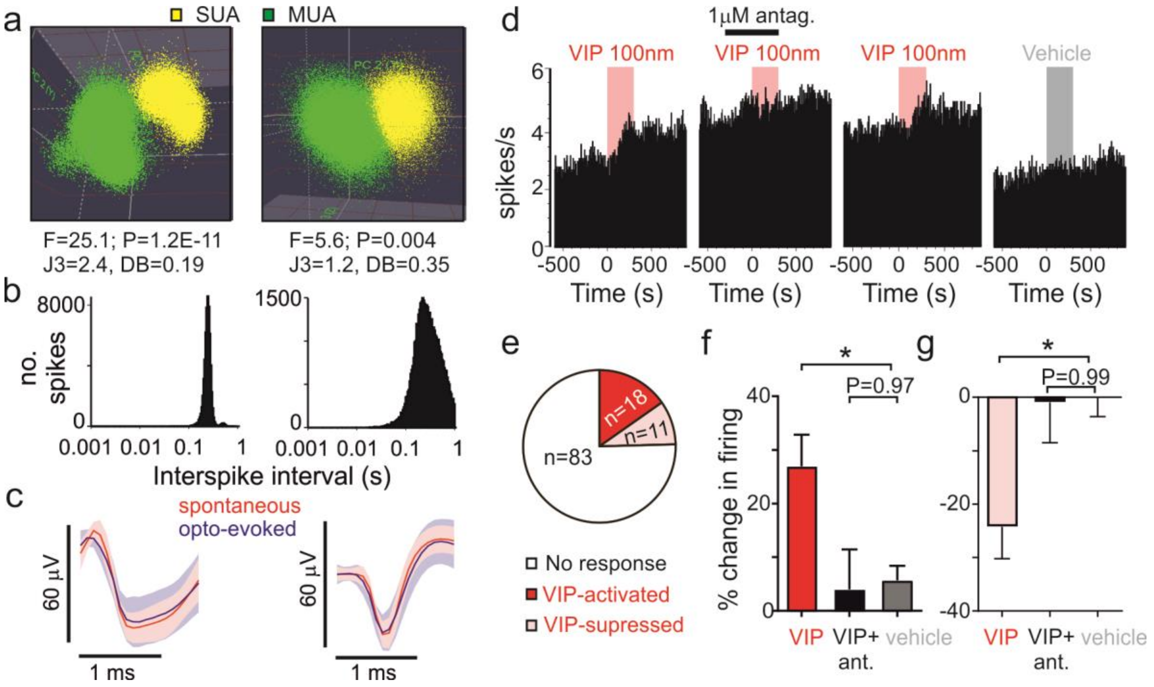

Supplementary Figure 2. Validation of VIP cell identification in VIP-ChR2 mice. (**a-c**) Principal component based clustering and sort-quality statistics (**a**; numbers beneath plots represent MANOVA F and P values, J3 and Davis Bouldin metrics for cluster separation), interspike interval histograms (**b**) and mean waveforms of spontaneous and optogenetically evoked spikes (**c**) for isolated VIP neurons in Supplementary Fig. 1e. (**d)** Representative SCN neuronal response to bath application of VIP (100nm; red shading) in the presence/absence of a VIP receptor antagonist and vehicle application (grey shading). (**e**) Proportions of neurons showing reproducible increases or decreases in firing following VIP application (from 4 slices). (**f**&**g**) Mean±SEM responses of VIP activated (**f**) and supressed (**g**) cells to application of VIP, VIP+antagonist and vehicle application. Data analysed by Data analysed by multilevel mixed effects linear model (**f**: $F_{2,26.22}$ = 5.66, P=0.01; **g**: $F_{2,19.67}$= 6.24, P=0.01) with Dunnett's post test. *=P<0.05.

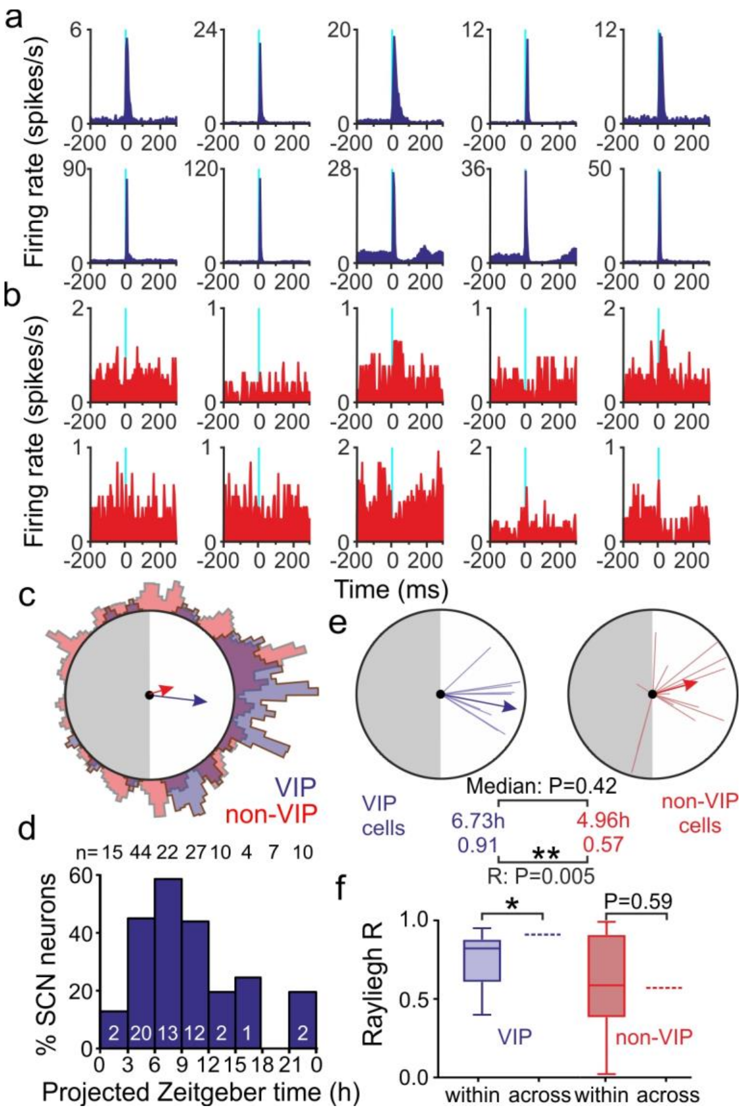

Supplementary Figure 3. Optogenetic identification of suprachiasmatic nucleus VIP neurons. (**a,b**) Histograms of spike responses to optogenetic pulses (10ms ramped; mean of ~1500 trials, timing of pulse indicated by vertical blue shading) for isolated VIP (**a**) and non-VIP (**b**) SCN neurons shown in Fig. 1c and d. (**c**) Overlaid Rayleigh plots (as in Fig. 1e) showing phase distribution for isolated VIP (blue) and non-VIP (red) SCN neurons that exhibited circadian variation in firing (n=52 & 87 cells respectively, from 16 slices, population density plots smoothed with Gaussian; σ=10min). (**d**) Distribution of peak firing times for VIP cells expressed as a percentage of the total number of isolated SCN neurons with peak firing falling in that epoch. Numbers of VIP cells in each bin are inset on each bar, total number of isolated SCN cells for each bin are displayed above. (**e**) Rayleigh vector plots of median phase and clustering strength for VIP and non-VIP SCN subpopulations across individual slices. Results from Rayleigh analysis on individual slices (those with at least 2 neurons/subpopulation that exhibited evidence of circadian variation; n=10 and 15 respectively for VIP and non VIP subpopulations) are shown by thin lines without arrowheads. Note that differences in phase and clustering strength (compared by Mann-Whitney U-test and Browne-Forsythe's tests respectively on linearised data) are qualitatively similar to those determined across the full population of VIP and non-VIP neurons (as illustrated in **c**). (**f**) Box and whisker plots showing estimates of clustering strength (Rayleigh R) for VIP and non-VIP subpopulations within individual slices and across the relevant group of slices, indicating that the variability of single cell phasing for subpopulations within slices is equivalent to (non-VIP) or greater (VIP) than that found between slices (one sample t-tests, P=0.59 and P=0.025 respectively).

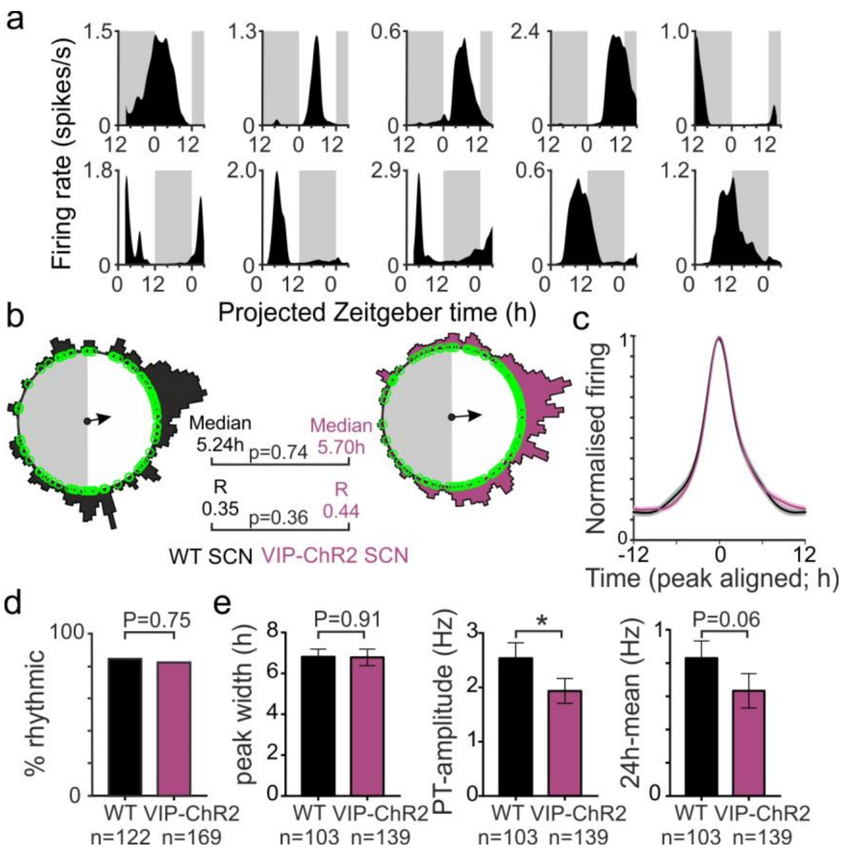

Supplementary Figure 4. Optogenetic cell identification does not disrupt SCN rhythmicity. (**a**) Example spontaneous firing activity plots from SCN neurons from in control (VIP$^{+/+}$; Ai32$^{+/-}$) brain slices prepared during late (upper panels) or early (lower panels) projected day. (**b**) Rayleigh plots showing phase distribution for isolated control and VIP-ChR2 (VIP$^{+/cre}$;Ai32$^{+/--}$) SCN neurons that exhibited evidence of circadian variation in firing (n=103 & 139 cells from 15 and 16 slices respectively). Individual cells are represented by open circles, external histograms represent relative population density (Gaussian smoothing; σ=10min). Phase and clustering strength compared by multilevel mixed-effects model and Browne-Forsythe's test respectively on linearised data. (**d**) Proportion of isolated control and VIP-ChR2 SCN neurons displaying evidence of circadian variation in firing, compared by Fishers exact test. (**e**) Peak width (full width at half-max firing; left), Peak-trough amplitude for firing rhythm (middle) and 24h mean firing rate (right) for rhythmic control and VIP-ChR2 SCN neurons. Data compared by multilevel mixed-effects linear models. (**f**) Normalised, peak aligned, 24h firing profiles of rhythmic VIP and non-VIP neurons.

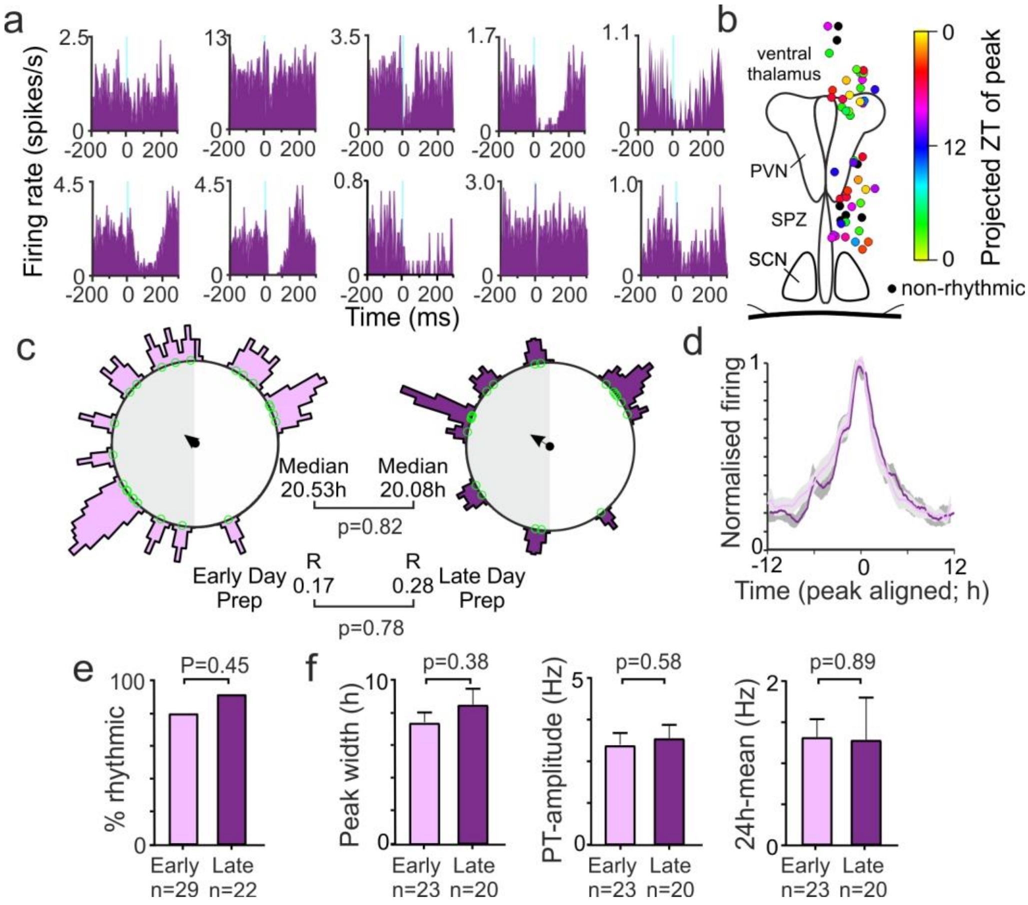

Supplementary Figure 5. Rhythmic properties of VIP^{in} cells do not vary according anatomical location or time of slice preparation. (**a**) Example firing rate histograms illustrating responses of VIP^{in} cells from Fig3a to optogenetic stimulation of SCN VIP neurons (10ms ramped pulse; means of ~1500 trials). (**b**) Anatomical locations of identified VIP^{in} cells colour coded according to projected ZT of peak firing. (**c**) Rayleigh plots showing phase distribution for isolated VIP^{in} cells from slices prepared during early and late projected day (n=23 and 20 rhythmic cells from 14 and 12 slices respectively). Conventions and analysis as in Fig 1e. (**d**) Mean±SEM normalised, peak aligned, 24h firing profiles of rhythmic VIP^{in} cells from early and late preparations. (**e**) Proportion of isolated VIP^{in} cells from early and late day preparations displaying evidence of circadian variation in firing (compared by Fishers exact test). (**f**) Peak width, Peak-trough amplitude (middle) and 24h mean firing rate (right) for VIP^{in} cells from early and late preparations, analysed by multilevel mixed-effects linear models.

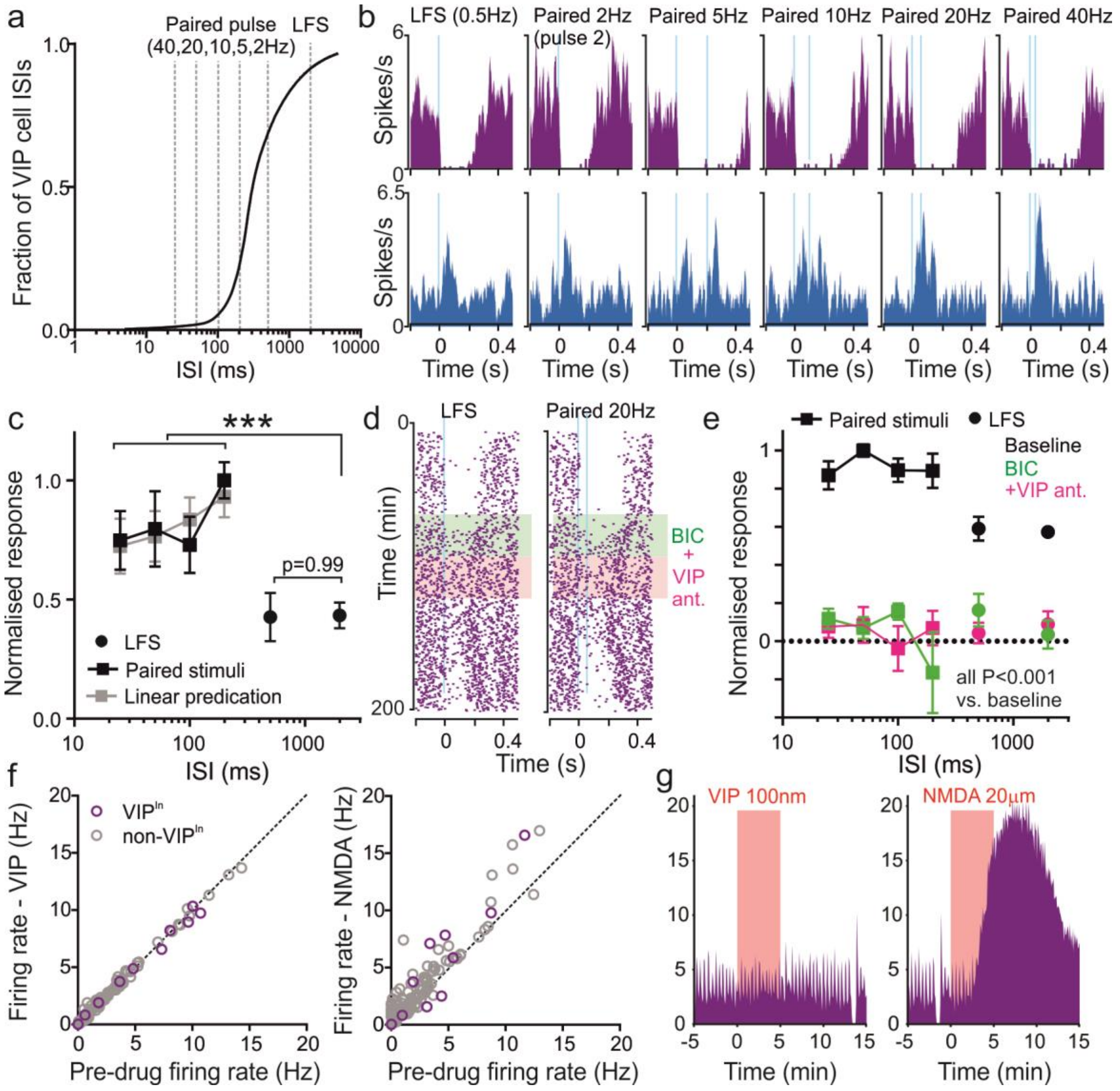

Supplementary Figure 6. Suprachiasmatic nucleus VIP neurons influence target cell firing rates via GABA not VIP-mediated signalling. (**a**) Cumulative interspike interval probability distribution for all VIP neurons (n=59) identified in the present study. (**b**) Peri-stimulus firing rate histograms for example VIP[in] (top) and cell with slow-activation (bottom) recorded *ex vivo* from the PVN and tested with paired optogenetic stimulation of the SCN (10ms flashes at 2-40Hz; 300 trials/stimulus). (**c**) Normalised mean±SEM responses to single (low frequency stimulation; LFS – 0.5Hz) and paired stimuli across the population (n=25 VIP[in] cells and n=3 cells with slow-activation), with predicted responses based on linear extrapolation from LFS data (see methods). Data were analysed by multilevel mixed-effects linear model (ISI: $F_{5,105.38}$=5.0, P<0.001; Actual vs. predicated: $F_{1,267.55}$=0.22, P=0.64; interaction with ISI: $F_{5,105.38}$=0.10, P=0.99) with Tukey's post-test. ***=P<0.001. (**d**) Peri-event rasters for representative VIP[in] cell responses to LFS and paired 20Hz optogenetic stimuli - prior to and following treatment with BIC and subsequent VIP antagonist co-application. (**e**) Normalised mean±SEM responses (from n=6 VIP[in] and n=3 cells with slow-activation) to single and paired stimuli pre- and post-antagonist treatment. Data were analysed by multilevel mixed effect linear model (ISI: $F_{5,23.378}$=1.31, P=0.29; Drug: $F_{2,8.96}$= 12.65, P=0.002; Drug X ISI: $F_{10,24.15}$=0.68, P=0.73) with Tukey's post-test. (**f**) Mean firing rates pre- and post-application of 100nm VIP (left) or 20μM NMDA (right) across a population of neurons recorded from VIP cell target regions (n=12 VIP[in] and n=157 non-VIP[in]). Note that VIP consistently evokes negligible changes in firing while many cells increase firing rate in response to NMDA. (**g**) Firing rate profile for an example VIP[in] cell showing no response to bath application of VIP but a vigorous response to NMDA treatment (confirming effective drug delivery to the neuron); red shaded regions indicate timing of drug application.

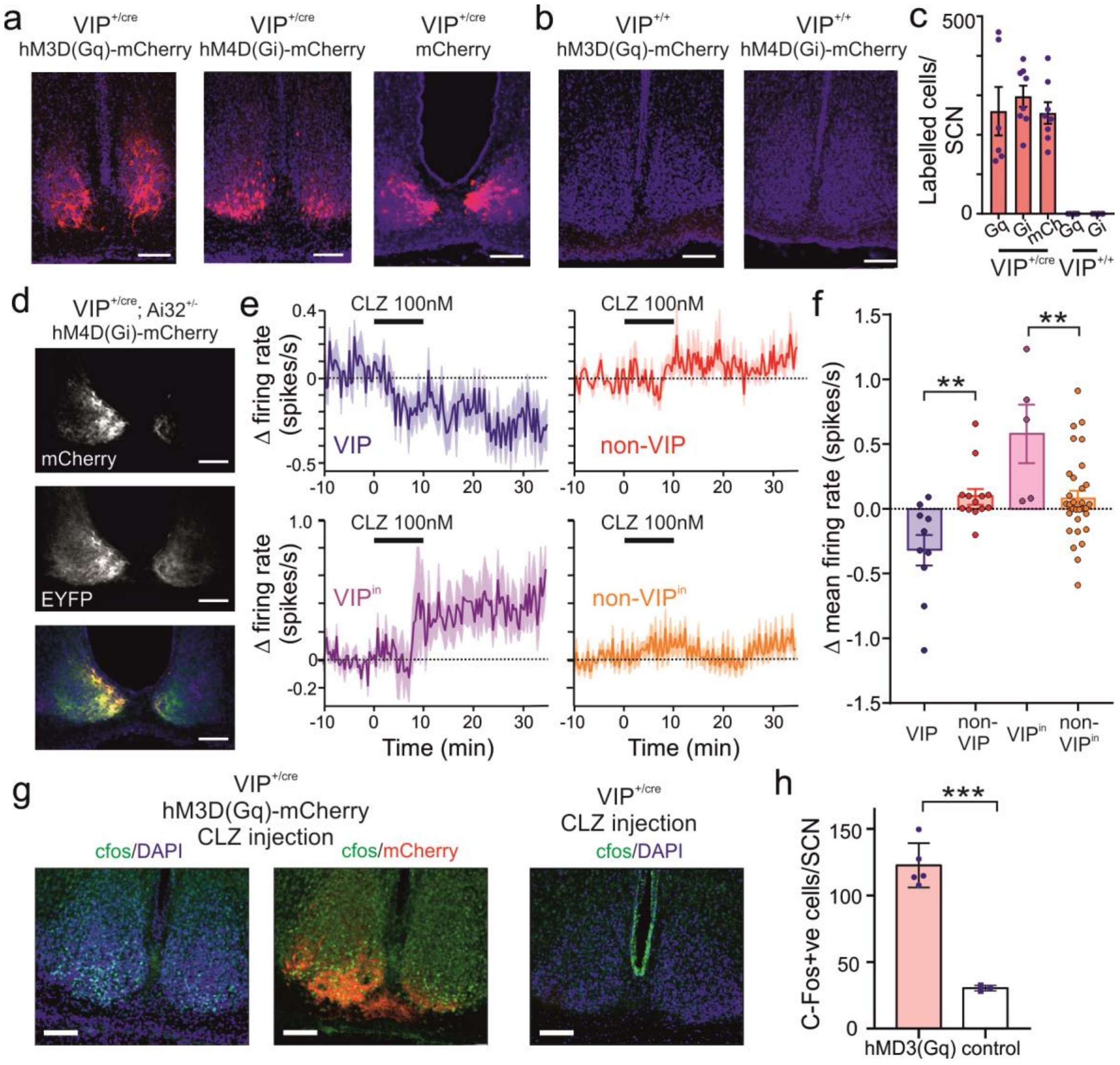

Supplementary Figure 7. Validation of DREADD-based manipulation of SCN VIP neurons. (**a**) Immuno-enhanced mCherry expression from representative VIP$^{+/cre}$ animals receiving intra-SCN microinjections of AAVs encoding activating (hMD3(Gq)) or inhibiting (hM4D(Gi)) DREADDs or control vector. Sections counterstained with DAPI. (**b**) As above but for viral injections in VIP$^{+/+}$ mice. (**c**) Quantification of mCherry labelled cells following viral injection in VIP$^{+/cre}$ and VIP$^{+/+}$ animals. No mCherry expressing cells were observed following injections in VIP$^{+/+}$ (n=4/group for Gq and Gi vectors). For injections in VIP$^{+/cre}$ the number of labelled cells/SCN was equivalent for each virus (n=6-8/group; One-way ANOVA, $F_{2,19}$=0.41, P=0.67). Across groups the mean number of labelled cells was 272±98 (mean±SD), corresponding to a transduction efficiency of 30±11% based on published SCN VIP cell counts (920 cells/SCN)[1]. (**d**) Endogenous hMD4(Gi)-mCherry and ChR2-EYFP expression from a representative virally transfected VIP$^{+/cre}$;Ai32$^{+/-}$ animal used for electrophysiological recordings. (**e**) Mean±SEM baseline-corrected firing rate over time relative to bath application of 100nm clozapine (CLZ) during mid-projected day for optogenetically identified cell populations detected in *ex vivo* recordings with penetrating multisite electrodes (n=10 SCN VIP, n=13 SCN non-VIP, n=5 extra-SCN VIP^{in} and n=31 extra-SCN non-VIP^{in}; from 9 slices). (**f**) Mean±SEM change in firing rate 15-35min post CLZ (relative to 10min pre-application) for cell populations in **e**. Data analysed by multilevel mixed-effects linear model ($F_{3,52.989}$=8.94, P<0.001) with Sidak's multiple comparisons. **=P<0.01. (**g**) Representative images of c-Fos immunoreactive neurons in hM3D(Gq)-mCherry expressing (right) and non-transfected VIP$^{+/cre}$ mice (left), 90min following CLZ (0.1mg/ml) injection at ZT14.5. (**h**) Quantification of c-Fos expressing cells in SCN from virally transfected and non-transfected mice as in **g** (n=4 and 3 respectively). Data analysed by unpaired t-test. ***=P<0.001. Scale bars throughout represent 100µm.

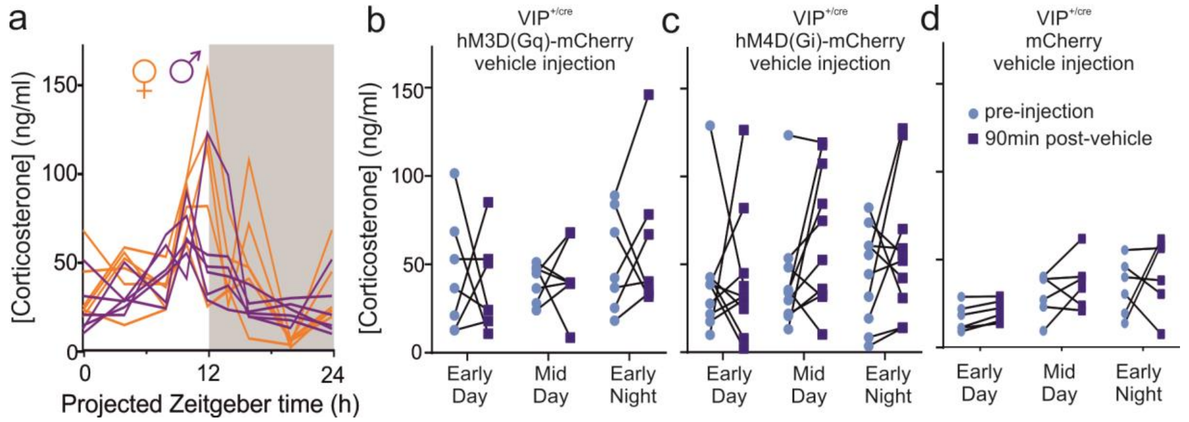

Supplementary Figure 8. Control corticosterone measurements. (**a**) Daily corticosterone concentration profiles for all Male (purple) and Female (orange) wild type mice contributing to Figure 6b. Analysis with two-way RM ANOVA indicated no significant effect of gender (**a**: Gender-$F_{1,10}$= 4.237, P=0.07; Time-$F_{8,80}$= 9.02, P<0.0001; Gender X Time – $F_{8,80}$=1.755; P=0.10) and so both male and female animals were used for DREADD experiments. (**b-d**) Circulating corticosterone concentration prior to and following i.p. injections of vehicle in Gq-DREADD (**b**; n=7), Gi-DREADD (**c**; n=10) and vector control (d; n=6 expressing animals (timings as indicated in Figure 6b). Analysis with two-way RM ANOVA indicated no significant effect of vehicle (**b**: vehicle- $F_{1, 6}$ = 0.06, P=0.81; Epoch- $F_{2, 12}$ = 1.42, P=0.28; Epoch X vehicle - $F_{2, 12}$ = 0.31, P=0.74; **c**: vehicle- $F_{1, 9}$ = 1.71, P=0.22; Epoch- $F_{2, 18}$ = 0.59, P=0.57; Epoch X vehicle - $F_{2, 18}$ = 0.89, P=0.43; **d**: vehicle- $F_{1, 5}$ = 1.63, P=0.26; Epoch- $F_{2, 10}$ = 5.04, P=0.03; Epoch X vehicle - $F_{2, 10}$ = 0.08, P=0.92).

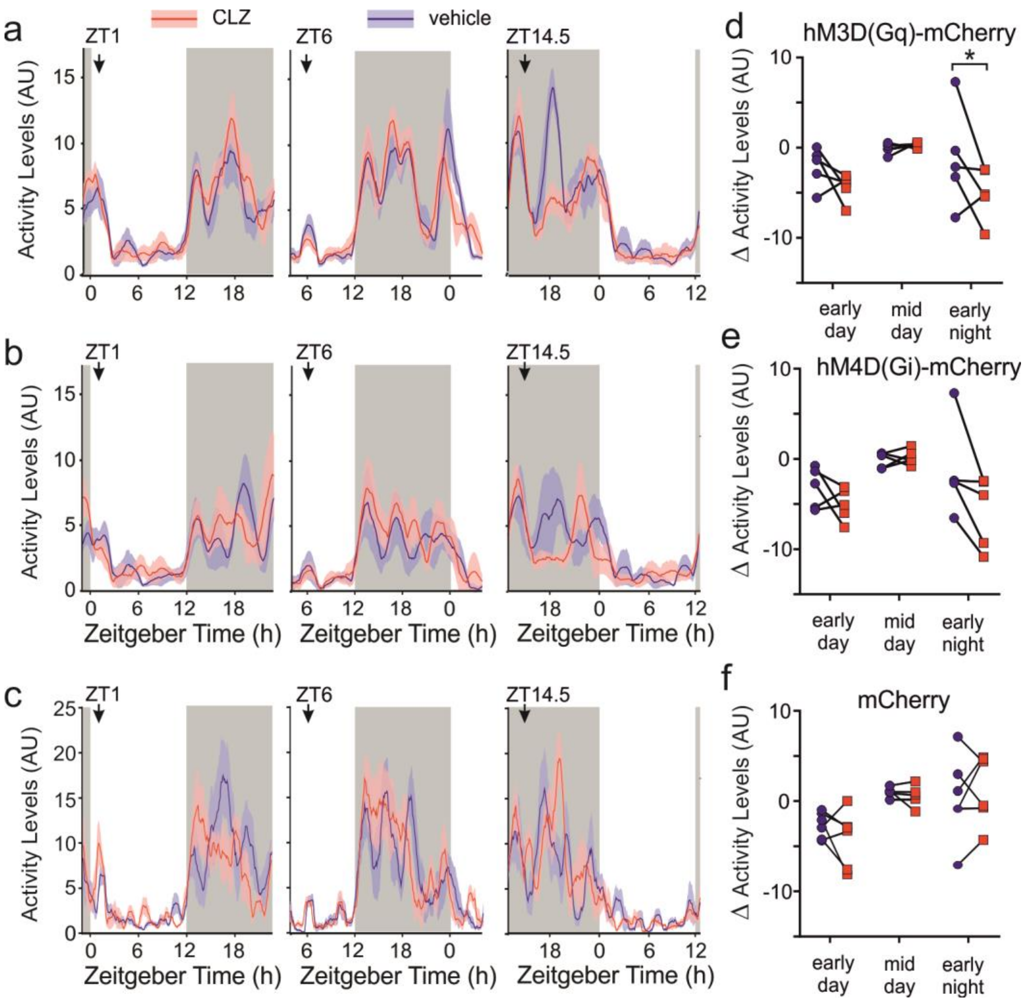

Supplementary Figure 9. Effect of chemogenetic manipulation of VIP cells on overall activity levels. (**a-c**) Mean activity level traces for all Gq-DREADD (**a**; n=5), Gi-DREADD (**b**; n=5) and control vector (**c**; n=6) expressing $VIP^{+/cre}$ animals for the 24 hour period surrounding i.p. injection of vehicle or clozapine (CLZ, 0.1mg/kg). Injection time points are indicated by arrows. (**d-e**) Changes in activity levels (Arbitrary Units; AU) following vehicle and CLZ injections in experimental groups as above. Data were analysed by two-way RM-ANOVA (**d**: CLZ- $F_{1,4}$ = 8.07, P=0.047; Epoch- $F_{2,8}$ = 5.72, P=0.029; Epoch X CLZ - $F_{2,8}$ = 2.0, P=0.106; **e**: CLZ- $F_{1,4}$ = 5.54, P=0.078; Epoch- $F_{2,8}$ = 3.77, P=0.07; Epoch X CLZ - $F_{2,8}$ = 1.83, P=0.22; **f**: CLZ- $F_{1,5}$ = 0.14, P=0.72; Epoch- $F_{2,10}$ = 4.51, P=0.04; Epoch X CLZ - $F_{2,10}$ = 1.24, P=0.33). Sidak's post-tests at individual epochs were performed on data form Gq-DREADD expressing animals (**c**; where ANOVA reported a significant main effect of CLZ). *=P<0.05.

Supplementary Reference

1 Lee, I. T. *et al.* Neuromedin s-producing neurons act as essential pacemakers in the suprachiasmatic nucleus to couple clock neurons and dictate circadian rhythms. *Neuron* **85**, 1086-1102 (2015).
